# Unveiling crucifer metabolomes *via* UPLC-HRMS/MS and chemometric analysis of edible and non-edible varieties

**DOI:** 10.1038/s41598-025-29178-w

**Published:** 2025-12-08

**Authors:** Mostafa H. Baky, Eman M. Kabbash, Ahmed Serag, Steffani Doll, Mohamed A. Farag

**Affiliations:** 1 Department of Pharmacognosy, Faculty of pharmacy, Egyptian Russian University, Badr city, 11829 Cairo Egypt; 2 Egyptian Drug Authority, Cairo, 11553 Egypt; 3 Pharmaceutical Analytical Chemistry Department, Faculty of Pharmacy, Al- Azhar University, Cairo, 11751 Egypt; 4 German Centre for Integrative Biodiversity Research (iDiv) Halle-Jena- Leipzig, 04103 Leipzig, Germany; 5 Pharmacognosy Department, College of Pharmacy, Cairo University, Cairo, 11562 Egypt

**Keywords:** Cruciferous vegetables, Glucosinolates, Phenolic compounds, UHPLC-HRMS/MS, Multivariate data analysis, Biochemistry, Plant sciences

## Abstract

Cruciferous green vegetables are considered as one of the most important consumed vegetables world-wide owing to their rich nutritive value, characteristic taste as well as health benefits. Cruciferous vegetables are rich in a myriad of bioactive phytochemicals including phenolic compounds and glucosinolates (GLS). The current work aims to assess heterogeneity in secondary metabolites profile among six cruciferous green leafy vegetables including *Brassica oleracea* (cabbage), *B. oleracea* var. *Italica* (broccoli), *B. oleracea* var. *oleracea* (cauliflower), *B. rapa* (turnip), *Raphanus sativus L.* (radish), and *Nasturtium officinale* (watercress) using UHPLC-HRMS/MS coupled with chemometric analysis. A total of 149 metabolites were identified belonging to different phytochemical classes including flavonoids, phenolic acids, steroids, anthocyanins, fatty acids/acyl esters, and GLS. Flavonoids were detected as the most abundant class in cruciferous vegetables represented by 48 peaks, especially Broccoli, cabbage and cauliflower. Radish exhibited unique flavonoid profiles including kaempferol-*O*-pentosylhexoside-*O*-deoxyhexoside, kaempferol-*O*-hexosyl-*O*-di-deoxyhexoside, and quercetin-*O*-tri-deoxyhexoside, along with indole-derived GLS known as methoxyspirobrassinin. Distinct phenolic acid profile was observed where di-sinapoyl-*O*-hexoside was detected only in turnip and broccoli, p-coumaroyl-malic acid was enriched in turnip only, and feruloyl malate and coumaroyl malate were enriched in turnip and radish. Unsupervised PCA analysis revealed distinct variation of turnip from other cruciferous samples, while OPLS-DA distinguished non-edible broccoli from edible cabbage, showing enrichment of neoglucobrassicin and malic acid in broccoli and higher flavonoid and anthocyanin levels in cabbage. These findings highlight the metabolic diversity of cruciferous leaves and support the valorization of underutilized species as potential functional food resources.

**Supplementary Information:**

The online version contains supplementary material available at 10.1038/s41598-025-29178-w.

## Introduction

A central question for consumers when selecting plant-based foods is whether a given plant part is commonly consumed or less frequently utilized. Recently, functional foods derived from natural sources catch the interest of both consumers and nutritional specialists as it plays a pivotal role in human diet not only because they are palatable but also due to their richness in several phytochemicals of both nutritional and health-promoting properties^1^. Such a revolution in functional foods manufacturing by role led to progress in quality assessment tools to ensure quality and identify new nutraceuticals. Green leafy vegetables are widely consumed worldwide either fresh or after processing or added to food recipes owing to their special taste and aroma, as well as their several health benefits^2^. Brassicaceae, also known as Cruciferae, is a well-known medium-sized flowering plant family that comprises ca. 360 genera, including 3709 species distributed worldwide^3^. Brassicaceae includes a wide variety of edible vegetables used widely in food owing to their nutritional value as being rich in vitamins (A, B1, B2, B6, C, E, and K) and minerals such as magnesium, iron, and calcium^3^. The most common vegetables in Brassicaceae include cabbage, cauliflower, broccoli, turnip, radish, watercress, and rocket^4^. Owing to their richness in a myriad of phyto, macro/micronutrients i.e., vitamins, carotenoids, dietary fibers, amino acids, minerals, glucosinolates (GLS), and phenolic compounds, brassica vegetables possess both nutritional and potential health value^5^. Brassica vegetables encompass a large class of biologically active secondary metabolites including GLS and other sulfur containing compounds^6,7^. GLS (*β*-thioglucoside-*N*-hydroxysulfates) structure is composed of a *β*-D-glycopyranose moiety attached by sulfur-linkage to a hydroximinosulfate ester with another amino group^8^. GLS possess several biological activities in addition to their culinary uses attributed to their unique flavor and taste^9^. Owing to their richness in GLS, cruciferous vegetables are attributed several positive effects including reducing the risk of cardiovascular disease, anticancer effect against lung, stomach, bladder, and colorectal^10^. Despite their nutritional richness, not all Brassicaceae leaves are equally consumed. For instance, cabbage, radish, and watercress leaves are commonly eaten, while the leaves of broccoli, cauliflower, and turnip are frequently discarded as by-products^7^. This disparity is largely related to sensory attributes (bitterness, fibrous texture), cultural dietary preferences, and, in some cases, higher levels of antinutritional compounds, despite the fact that these leaves remain edible and potentially valuable sources of nutrients and bioactives^2^.

Cabbage (*Brassica oleracea*) is one of the most consumed edible cruciferous leafy vegetable worldwide owing to its special taste and richness in antioxidant phytochemicals^11^. Broccoli (*Brassica oleracea* var. *italica*) is likewise nutritionally important vegetable for its richness in vitamins A, B2, and C, minerals, in addition to phytonutrients such as polyphenols, *β*-carotene and α-tocopherol, and isothiocyanates^12^. Cauliflower (*Brassica oleracea* var. *oleracea*) is rich of antioxidants phytonutrients such as GLS, ascorbic acid and polyphenols^13^. Turnip (*Brassica rapa*) is a widely consumed brassica rich in GLS, flavonoids, indoles, phenolics, and essential oil^14^. Radish (*Raphanus sativus* L.) leafy part is typically consumed green to encompass several chemicals including vitamins, carbohydrates, minerals, flavonoids, and GLS^15^. Watercress (*Nasturtium officinale*) is an important member of brassica vegetables rich in phytochemicals specially GLS^16^.

Ensuring the quality of plant-based functional foods, including chemical attributes, is important to assess their food or nutraceutical value based on chemical composition^17^. Recently, metabolomics represents an important platform used to characterize the quality of food-stuff using different spectroscopic techniques such as mass spectrometry based chromatographic techniques^18^. Ultra-high performance liquid chromatography coupled with high resolution mass analyzer (UPLC-MS/MS) is a platform well suited for profiling secondary metabolites. We have previously reported on the application of MS-based metabolomics in aroma and nutrients profiling in cruciferous leafy vegetables *via* gas chromatography mass spectrometry (GC-MS) in context to edible and non-edible plants^7^. Compared to GC-MS that targets aroma compounds, UPLC-MS/MS technique is more suited for large molecular weight polar metabolites that account for health benefits^19,20^. Distribution of secondary metabolites among leafy green crucifers in the context of edible or nonedible has yet to be reported, especially targeting its GLS composition. Further application of multivariate data analysis including unsupervised principal component analysis (PCA) and supervised orthogonal projection to latent structures discriminant analysis (OPLS-DA) were employed for samples’ classification. Compared with PCA, OPLS-DA has a greater capacity for marker recognition and discrimination among samples by supplying the most pertinent variables for the distinction among two class groups^2^.

To date, the distribution of secondary metabolites among cruciferous leafy vegetables has not been systematically reported in relation to consumption practices, particularly regarding GLSs and phenolic compounds. Here, we employed UHPLC-HRMS/MS combined with multivariate data analysis, including PCA and supervised OPLS-DA, to assess metabolic heterogeneity among six cruciferous leafy vegetables three commonly consumed (cabbage, radish, watercress) and three less commonly consumed (broccoli, cauliflower, turnip) collected from the same origin. The main goal of this study was to investigate the heterogeneity of secondary metabolites in these six cruciferous leafy vegetables, with emphasis on GLSs and phenolic compounds, and to highlight the nutritional and potential health relevance of underutilized leafy parts. Such comprehensive profiling may support the valorization of agricultural by-products and contribute to their future use in functional foods and nutraceutical development.

## Results & discussion

### Metabolites identification in cruciferous leaves *via* UPLC-MS/MS

**Fig. 1:**
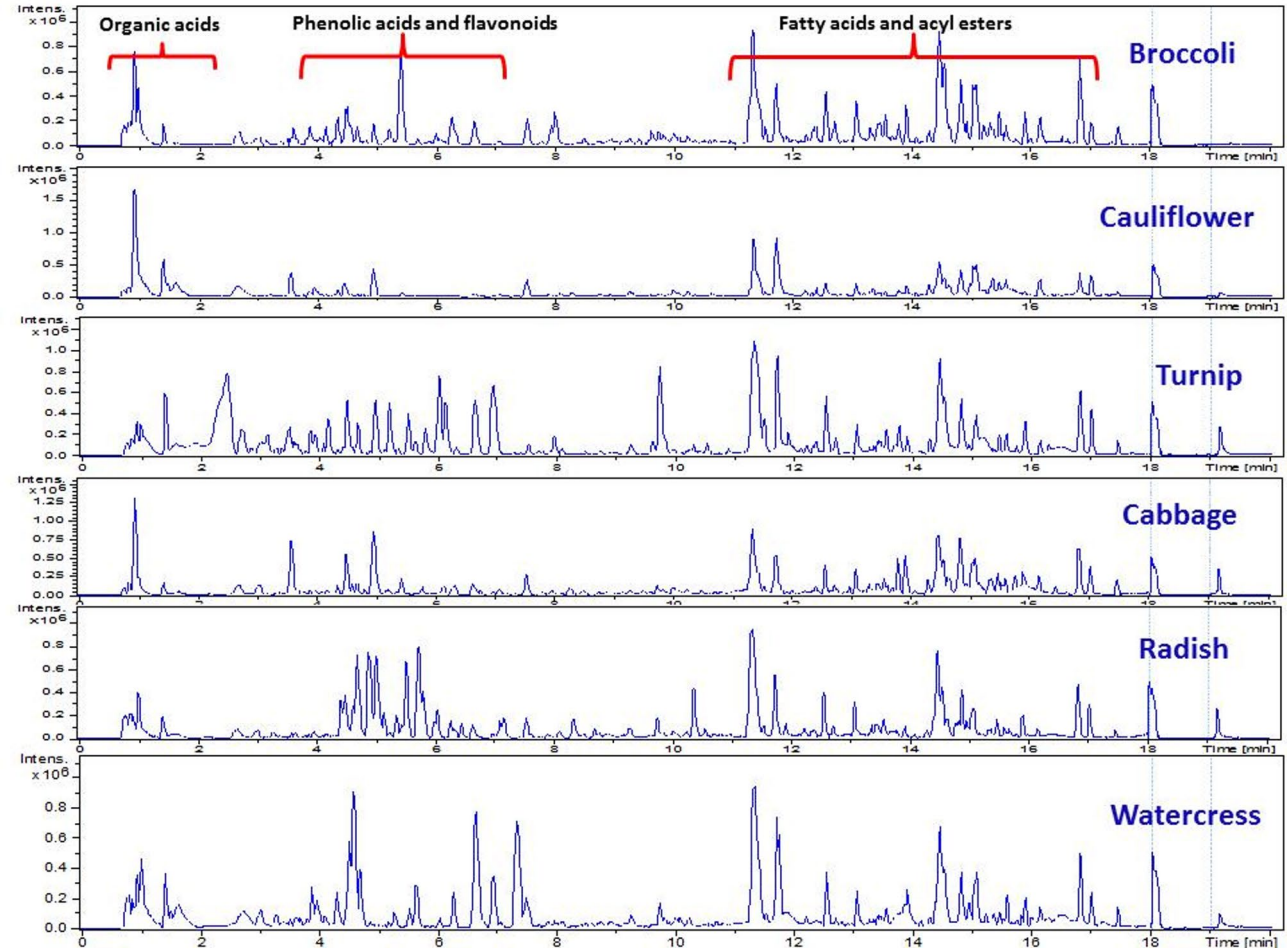
Representative UPLC-MS/MS chromatogram of cruciferous leaves methanol extracts traces analyzed in the negative ion mode.

**Fig. 2:**
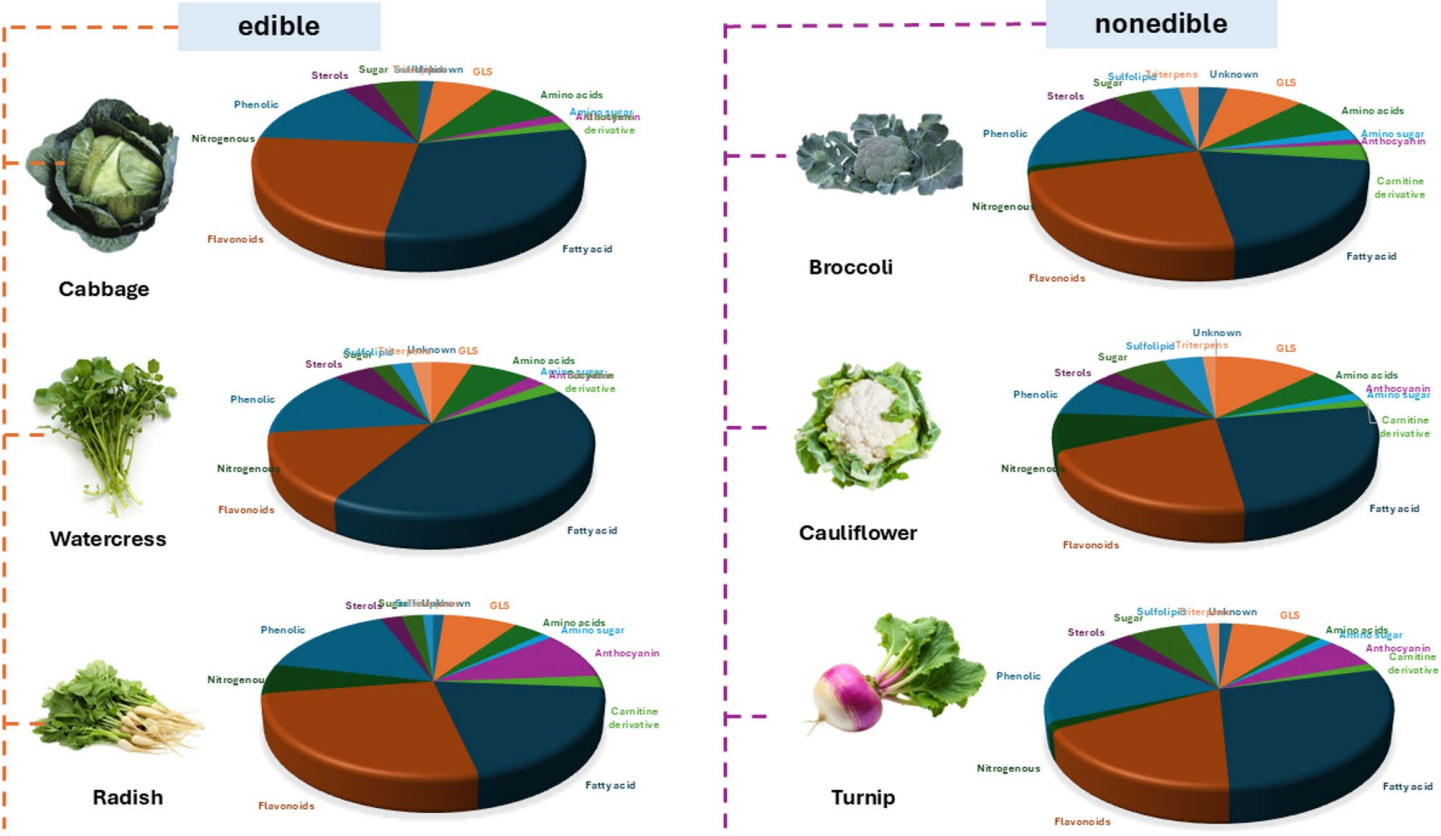
Pie charts representing the major classes identified in both edible and nonedible cruciferous leaves, the charts were generated based on the number of peaks identified for each class.

UPLC-MS/MS analysis led to the annotation of 149 metabolites in the examined cruciferous vegetables based on accurate mass, fragmentation patterns, comparison with literature, and in-house database searching. The derived base peak chromatograms for different extracts showed both qualitative and quantitative differences among the examined crucifers. Analysis was carried out in both negative (Fig. 1) and positive (Suppl. **Fig. ****S1**) ionization modes, providing greater coverage of the crucifer metabolome belonging to different classes including glucosinolates (GLS) (15 Peaks), phenolic acids (16 peaks), flavonoids (42 peaks), anthocyanidins (9 peaks), amino acids (10 peaks), fatty acids/fatty acyl glycosides (20 peaks), sulfolipids (3 peaks), and sterols (4 peaks), in addition to a number of identified metabolites belonging to different classes (Fig. 2). Due to their sulfate glucose core, Brassicaceae-specific glucosinolates ionize more readily in negative electrospray ionization mode (ESI) and can be better detected^20^. Also, secondary metabolites such as phenylpropanoids and flavonoids can be more readily detected in negative mode due to their relatively acidic nature. In contrast, metabolites showed improved detection in positive mode including polyamines, alkaloids, anthocyanins, and sterols, warranting the value of employing both modes in detection. Metabolite identification was based on their molecular formula, elution order, and main fragment ions in comparison to previously reported data (Table 1) and is detailed in the next subsections for each metabolite class.

**Table 1:** Identification of secondary metabolites in cruciferous vegetables detected in UPLC-MS/MS analysis in both positive and negative mode.

No	Rt min	Compound name	Chemical class	[M-H]^−^/ [M + H]^+^	Neutral Molecular formula	Neutral Molecular formula	error	MS/MS fragments	References	Broccoli	cauliflower	Cabbage	turnip	Radish	Watercress
1.	0.94	Hexose	Sugar	**[M − H]**^-^ 179.0569	C_6_H_12_O_6_	C_6_H_12_O_6_	−4.2	161.0471	^43^	+	+	+	+	**+**	**+**
2.	0.952	Gluconic acid	Organic acid	**[M − H]**^-^ 195.0507	C_6_H_11_O_7_^−^	C_6_H_12_O_7_	1.5	75.0091 129.0197	^22^	+	+	+	+	+	+
3.	0.961	Glutamine	Amino acid	[M + H]⁺ 147.0764	C_5_H_11_N_2_O_3_^+^	C_5_H_10_N_2_O_3_	−9.3	130.0500 102.0550 85.0480	^44^	+	+	+	-	+	+
4.	0.965	Unknown	Sugar	[M − H]⁻ 307.1173	C_12_H_19_O_9_^−^	C_12_H_20_O_9_	−2.3	289.1063 145.0603		+	+	-	+	+	-
5.	0.966	Methoxygallic acid hexoside	Phenolic acid	[M + H]⁺ 365.1053	C_14_H_21_O_11_^+^	C_14_H_20_O_11_	8.0	203.0528 175.0192 130.0499		+	-	-	-	-	-
6.	0.967	Trisaccharide	Sugar	[M − H]⁻ 539.1396	C_24_H_27_O_14_^−^	C_24_H_28_O_14_	1.9	377.0870 215.0336 195.0515 179.0567		+	+	+	+	-	-
7.	0.969	Glutamic acid	Amino acid	[M − H]⁻ 146.0456	C_5_H_8_NO_4_^−^	C_5_H_9_NO_4_	2.1	102.0559	^44^	+	-	+	-	+	-
8.	0.986	Disaccharide	Sugar	[M − H]⁻ 377.0874	C_18_H_17_O_8_^−^	C_18_H_18_O_8_	6.9	215.0338 195.0519		+	+	+	+	-	-
9.	1.020	Quinic acid	Phenolic acid	[M − H]⁻ 191.0566	C_7_H_11_O_6_^−^	C_7_H_12_O_6_	−2.5	173.0119 111.0008		+	+	+	+	+	+
10.	1.036	Malic acid	Phenolic acid	[M − H]⁻ 133.0133	C_4_H_5_O_5_^−^	C_4_H_6_O_5_^−^	1.1	115.0031 71.0135		+	+	+	+	+	+
11.	1.048	Tyrosine	Amino acid	[M + H]⁺ 182.0814	C_9_H_12_NO_3_^+^	C_9_H_11_NO_3_	2.3	164.0706 120.0813		+	+	+	+	+	+
12.	1.051	Methylmalonylcarnitine	Carnitine derivative	[M + H]⁺ 262.1285	C_11_H_20_NO_6_^+^	C_11_H_19_NO_6_	−2.3	244.1169 174.0395		+	-	-	-	+	-
13.	1.050	Citric acid	Organic acid	[M − H]⁻ 191.0186	C_6_H_7_O_7_^−^	C_6_H_8_O_7_	6.6	173.0141 129.0192 111.0078		+	+	-	-	+	+
14.	1.053	Glucoiberin (3-Methylsulphinylpropyl glucosinolate)	Aliphatic Glucosinolate	[M − H]⁻ 422.0228	C_11_H_19_NO_10_S_3_^−^	C_11_H_20_NO_10_S_3_	6.3	358.0237 290.0859 195.0123 96.6577	^33^	-	-	+	+	-	-
15.	1.07	Gluconapin	Aliphatic Glucosinolate	[M − H]⁻ 372.0422	C_11_H_18_NO_9_S_2_^−^	C_11_H_19_NO_9_S_2_	1.8	290.087 243.0619 195.057	^45^	-	-	-	+	-	-
16.	1.10	Glucoraphanin	Aliphatic Glucosinolate	[M − H]⁻ 436.0428	C_12_H_22_NO_10_S_3_^−^	C_12_H_23_NO_10_S_3_	−3.9	243.0636 195.0518 133.0145	^45^	+	++	-	-	+	++
17.	1.434	Glucoraphenin	Aliphatic Glucosinolate	[M − H]⁻ 434.02552	C_12_H_20_NO_10_S_3_^−^	C_12_H_21_NO_10_S_3_	−1.6	259.0149 243.0636 191.0198	^46,47]^	-	-	-	-	+	-
18.	1.455	Fructosyl-valine	Amino acid	280.1387	C_11_H_22_NO_7_^+^	C_11_H_21_NO_7_	1.2	262.1282 244.1174 216.1229		+	-	-	-	-	-
19.	1.479	Sinigrin	Glucosinolate	[M − H]⁻ 358.1987	C_10_H_16_NO_9_S_2_^−^	C_10_H_17_NO_9_S_2_	−1.3	243.0603 191.0182	^48^	+	+	-	-	+	-
20.	1.537	Isoleucyl-*O*-hexoside	Amino acid	[M − H]⁻ 292.1386 [M + H]⁺ 294.1540	C_12_H_22_NO_7_^−^ C_12_H_24_NO_7_^+^	C_12_H_23_NO_7_	5.6 2.3	202.1064 130.0867**/** 132.1020		+	+	+	-	-	-
21.	1.543	O-Glutarylcarnitine	Carnitine derivative	[M + H]⁺ 276.1432	C_12_H_22_NO_6_^+^	C_12_H_21_NO_6_	3.7	258.1325 230.1379	^49^	+	+	+	+	+	+
22.	1.727	Hydroxybutyrylcarnitine	Carnitine derivative	[M + H]⁺ 248.1487	C_11_H_22_NO_5_^+^	C_11_H_21_NO_5_	2.4	230.1450 212.0889	^49^	+	-	-	-	-	-
23.	2.033	N-acetyl hexosamine	Amino sugar	[M − H]⁻ 222.0619	C_7_H_12_NO_7_^−^	C_7_H_13_NO_7_	0.3	178.0734		+	-	-	-	-	-
24.	2.038	Hexosamine	Amino sugar	[M − H]⁻ 178.0738	C_6_H_12_NO_5_^−^	C_6_H_13_NO_5_	−9.7	160.0632 142.0526		+	-	-	-	-	-
25.	2.475	Unknown	Amino sugar	[M − H]⁻ 424.1427	C_16_H_26_NO_12_^−^	C_16_H_27_NO_12_	7.9	262.0924		-	+	-	++	+	-
26.	2.57	Hexose phenylalanine	Amino acid	326.1232 [M + H]⁺ 328.1384	C_15_H_20_NO_7_^−^ C_15_H_22_NO_7_^+^	C_15_H_21_NO_7_	3.9 5.0	164.0703/ 310.1278 166.0862		+	+	+	-	-	+
27.	2.64	Phenyl alanine	Amino acid	[M − H]⁻ 164.0717	C_9_H_10_NO_2_^−^	C_9_H_11_NO_2_	−6.5	147.0463 106.0550		+	-	-	-	-	-
28. **-**	2.72	Cinnamic acid	Phenolic acid	[M − H]⁻ 147.0456	C_9_H_7_O_2_^−^	C_9_H_8_O_2_	−2.9	103.0698		+	-	-	-	+	-
29.	2.723	Leucyl-Glutamine	Dipeptide	[M + H]⁺ 260.1591	C_11_H_22_N_3_O_4_^+^	C_11_H_21_N_3_O_4_	3.9	242.1495 197.1277		+	-	-	+	-	-
30.	2.739	Protocatechuic-acid hexoside	Phenolic compound	[M − H]⁻ 203.1393	C₁₃H₁_5_O₉^−^	C₁₃H₁₆O₉	−0.3	153.0181		-	-	-	+	+	-
31.	2.96	Dihydrogluconapin (Methylpropylglucosinolate)	Glucosinolate	[M − H]⁻ 374.0588	C_11_H_20_NO_9_S_2_^−^	C_11_H_21_NO_9_S_2_	−0.8	259.1464 244.1305 164.0723	^50^	-	-	-	+	-	-
32.	2.971	Gentisic acid*-O-* hexoside	Phenolic acid	[M − H]⁻ 315.0737	C_13_H_15_O_9_^−^	C_13_H_16_O_9_	−5.1	153.0194		+	-	+	-	+	+
33.	3.08	Vanillicacid*-O-*hexoside	Phenolic acid	[M − H]⁻ 329.0884	C_14_H_17_O_9_^−^	C_14_H_18_O_9_	−1.8	167.0351		+	-	-	+	+	-
34.	3.159	Unknown	-	[M − H]⁻ 259.1308	C_16_H_19_O_3_^−^	C_16_H_20_O_3_	5.0	241.1186 197.1298 171.1484		+	-	-	-	-	-
35.	3.452	Pentenylglucosinolate (Glucobrassicanapin)	Glucosinolate	[M − H]⁻ 386.0585	C_12_H_20_NO_9_S_2_^−^	C_12_H_21_NO_9_S_2_	−0.0	195.0344 96.9591	^50^	+	+	+	-	-	+
36.	3.461	Quercetin-*O*-caffeoyldeoxyhexosyl–tri-*O*- hexoside	Flavonoid	[M − H]⁻ 1095.2897	C_48_H_55_O_29_^−^	C_48_H_56_O_29_	−10.3	933.2426 787.1872 463.0440 301.0770	^51^	+	+	+	-	-	-
37.	3.521	Nicotinic acid-O-pentoside	Nitrogenous	[M − H]⁻ 254.0663	C_11_H_12_NO_6_^−^	C_11_H_13_NO_6_	2.9	124.0392		-	+	-	+	-	-
38.	3.526	Quercetin-*O*-sinapoylhexoside-*O*-deoxyhexoside	Flavonoid	[M − H]⁻ 817.2055	C_38_H_41_O_20_^−^	C_38_H_42_O_20_	−0.6	609.1470 447.0918		+	+	+	+	-	-
39.	3.546	Cyanidin-*O*-tetra-hexoside	Anthocyanin	[M + H]⁺ 935.2672	C_39_H_51_O_26_^+^	C_39_H_50_O_26_	1.9	773.1220 611.1612 449.1077 287.0546		+	-	+	-	-	-
40.	3.62	Kaempferol-*O*- dihexoside –di-*O-*hexoside.	Flavonoid	[M − H]⁻ 933.2563	C_39_H_49_O_26_^−^	C_39_H_50_O_26_	−1.8	771.1999 609.1450 489.1227 285.0323	^52^	+	+	+	-	-	-
41.	3.65	Quercetin-*O*-dihexoside -*O*-hexosyldeoxyhexoside	Flavonoid	[M − H]⁻ 933.2478	C_39_H_49_O_26_^−^	C_39_H_50_O_26_	−2.1	771.1948 447.0349 301.1754	^53^	+	+	+	-	-	-
42.	3.67	Kaempferol-*O*-sinapoylcaffeoyl–tri*-O*-hexoside	Flavonoid	[M − H]⁻ 1139.2974	C_53_H_55_O_28_^−^	C_53_H_56_O_28_	−7.8	933.2480 771.1925 609.1419 229.1541		-	-	+	-	-	-
43.	3.682	Leucyl-leucyl-glutamic acid	Peptide	[M + H]⁺ 374.2283	C_17_H_32_N_3_O_6_^+^	C_17_H_31_N_3_O_6_	0.7	261.1440 227.175		-	-	-	-	+	-
44.	3.69	Unknown	-	[M − H]⁻ 229.1584	C_16_H_21_O^−^	C_16_H_22_O^−^	5.9	211.1462 185.1673 130.877		+	-	-	+	-	-
45.	3.782	kaempferol-*O*-trihexoside	Phenolic acid	[M − H]⁻ 591.1721	C_24_H_31_O_17_^−^	C_24_H_32_O_17_^−^	−0.3	545.1543 383.1006	^54^	+	-	-	-	-	-
46.	3.828	Unknown	-	[M − H]⁻ 525.1602	C_24_H_29_O_13_ -	C_24_H_30_O_13_	2.2	363.1034		-	-	-	-	+	-
47.	3.84	Kaempferol-*O*-(methoxycaffeoyl) pentoside-*O*- trihexoside	Flavonoid	[M − H]⁻ 1095.2859	C_48_H_55_O_29_^−^	C_48_H_56_O_29_	−2.2	963.2418 771.1985 287.1570	^55^	-	-	-	+	-	-
48.	3.858	Quercetin-*O*- methoxycaffeoyldeoxyhexoside dihexoside-*O*-pentoside	Flavonoid	[M − H]⁻ 1095.2817	C_48_H_55_O_29_^−^	C_48_H_56_O_29_	1.3	771.1987 625.1407	^56^	-	-	-	+	-	-
49.	3.98	Kaempferol-*O*- hydroxyferuloyl-tri-*O*-hexoside	Flavonoid	[M − H]⁻ 963.2435	C_43_H_47_O_25_^−^	C_43_H_48_O_25_	−2.4	639.1725 447.0524 285.1777		+	-	-	-	-	-
50.	4.001	Glucobrassicin	Glucosinolate	[M − H]⁻ 447.0535	C_16_H_19_N_2_O_9_S_2_^−^	C_16_H_20_N_2_O_9_S_2_	−1.8	179.0574 96.9590	^57^	+	+	+	+	+	-
51.	4.083	Kaempferol- *O*-sinapoyl- tri -hexosyl- *O*-pentoside	Flavonoid	[M − H]⁻ 1109.3011	C_49_H_57_O_29_^−^	C_49_H_58_O_29_	−1.8	977.2579 771.2009 609.1472 285.1264		+	-	+	+	-	+
52.	4.102	Isorhamnetin-*O* caffeoylpentosyl deoxyhexosyl di-*O*-hexoside	Flavonoid	[M − H]⁻ 1079.2865	C_48_H_55_O_28_^−^	C_48_H_56_O_28_	7.9	771.1951 609.1242 316.1858		-	+	+	-	-	-
53.	4.13	Lariciresinol-O-dihexoside	Lignan	[M − H]⁻ 683.2488	C_32_H_43_O_16_^−^	C_32_H_44_O_16_	−1.4	521.2043 359.1515	^58^	+	-	-	-	-	-
54.	4.185	Leucyl-leucine	Peptide	[M − H]⁻ 245.1854	C_12_H_25_N_2_O_3_^−^	C_12_H_26_N_2_O_3_	2.2	199.1803 132.1008		+	-	-	-	-	-
55.	4.207	Kaempferol-*O-* methoxycaffeoyldeoxyhexoside tri- *O*-hexoside	Flavonoid	[M − H]⁻ 947.2477	C_43_H_47_O_24_^−^	C_43_H_48_O_24_	−0.5	917.2372 591.2667 287.1776		+	-	+	-	-	-
56.	4.212	Unknown	Nitrogenous	[M − H]⁻ 452.1662	C_14_H_30_NO_15_^−^	C_14_H_31_NO_15_	−8.9	362.1349 290.1131 184.1002 179.0553	^59^	-	+	-	-	+	-
57.	4.250	Kaempferol-*O*- feruloyldihexoside-*O*-hexoside	Flavonoid	[M − H]⁻ 947.2477	C_43_H_47_O_24_^−^	C_43_H_48_O_24_	−1.7	609.1419 286.1752		+	-	-	-	-	-
58.	4.260	Methoxytyrosine-*O*-hexoside	Nitrogenous	[M − H]⁻ 372.1271	C_16_H_22_NO_9_^−^	C_16_H_23_NO_9_	7.9	328.1058 210.0751		-	+	-	-	+	-
59.	4.263	Unknown flavonoid glycoside	Flavonoid	[M − H]⁻ 917.2363	C_42_H_45_O_23_^−^	C_42_H_46_O_23_	6.9	771.2002 593.2445 315.0755		+	+	+	-	+	-
60.	4.270	Unknown	Nitrogenous	[M − H]⁻ 474.2150	C_25_H_32_NO_8_^−^	C_25_H_33_NO_8_	5.9	384.1751 312.1545		-	+	-	-	+	-
61.	4.277	Feruloyl-*O*-hexoside	Phenolic acid	[M − H]⁻ 355.1042	C_16_H_19_O_9_^−^	C_16_H_20_O_9_	−2.2	193.0520		+	+	+	+	+	+
62.	4.288	Delphinidin-*O*-deoxyhexoside-di-*O*-hexoside	Anthocyanin	[M + H]⁺ 773.2213	C_33_H_41_O_21_^+^	C_33_H_40_O_21_	1.4	611.1610 449.1077 303.0494	^60^	-	-	-	-	+	+
63.	4.290	Hydroxyglucobrassicin	Glucosinolate	[M − H]⁻ 463.1705	C_16_H_19_N_2_O_10_S_2_^−^	C_16_H_20_N_2_O_10_S_2_	5.6	283.1907 224.0989	^57^	++	+	-	-	-	-
64.	4.303	(Methylsulfonyl)octylglucosinolate	Glucosinolate	[M − H]⁻ 508.0958	C_16_H_30_N_2_O_11_S_3_^−^	C_16_H_31_N_2_O_11_S_3_	5.6	464.1060 266.0862	^61^	-	-	-	-	+	-
65.	4.380	Methylsulfinyloctyl-glucosinolate (Glucohirsutin)	Glucosinolate	[M − H]⁻ 492.0462	C_16_H_30_NO_10_S_3_^−^	C_16_H_31_NO_10_S_3_^−^	−0.7	411.0462 259.0513		+	-	+	-	-	-
66.	4.399	Kaempferol-*O*-pentosyldeoxyhexoside-di-*O*-hexoside	Flavonoid	[M − H]⁻ 887.2451	C_38_H_47_O_24_^−^	C_38_H_48_O_24_	1.2	755.2030 593.1496 433.2074 431.1582		-	-	-	-	+	-
67.	4.427	Quercetin-*O*-feruloyl-*O*-di-pentoside	Flavonoid	[M − H]⁻ 741.1876	C_35_H_33_O_18_^−^	C_35_H_34_O_18_	10.0	609.1361 433.0756		-	-	-	-	+	-
68.	4.420	Gluconasturtiin	Glucosinolate	[M − H]⁻ 422.0596	C_15_H_20_NO_9_S_2_^−^	C_15_H_21_NO_9_S_2_	−2.7	342.1027 404.0490 216.0169		-	+	-	+	-	-
69.	4.440	Sinapoyl-*O*-hexoside (Raphanusol B)	Sinapic acid derivative	[M − H]⁻ 385.1133	C_17_H_21_O_10_^−^	C_17_H_22_O_10_	−1.9	223.0560		+	+	+	+	+	+
70.	4.443	Unknown	Unknown	[M − H]⁻ 439.21202	C_26_H_31_O_6_^−^	C_26_H_32_O_6_	5.6	349.1752 277.1576		-	-	-	-	+	+
71.	4.488	Kaempferol- *O*-hexosyl-*O*-deoxyhexosylhexoside	Flavonoid	[M − H]⁻ 755.2037	C_33_H_39_O_20_^−^	C_33_H_40_O_20_	2.1	593.1507 447.0935 285.0431		-	-	-	-	+	+
72.	4.491	Delphinidin di- *O* -hexosidedi -*O*- deoxyhexoside	Anthocyanin	[M + H]⁺ 919.2750	C_39_H_51_O_25_^+^	C_39_H_50_O_25_	−3.9	757.2183 611.1595 304.1743		-	-	-	+	+	-
73.	4.493	Methoxytyrosin	Nitrogenous	[M − H]⁻ 210.0787	C_10_H_12_NO_4_^−^	C_10_H_13_NO_4_	−3.8	124.0412		-	+	-	-	+	-
74.	4.520	Quercetin di-*O*-hexoside	Flavonoid	[M − H]⁻ 625.1367	C_27_H_29_O_17_^−^	C_27_H_30_O_17_	6.9	463.0878 301.0336		+	-	-	-	+	+
75.	4.555	Kaempferol-*O*-pentosylhexoside-*O*-deoxyhexoside	Flavonoid	[M − H]⁻ 725.1937	C_32_H_37_O_19_^−^	C_32_H_38_O_19_	−0.3	579.1279 417.0823		-	-	-	-	+	-
76.	4.560	Glucoarabin (methylsulfinyl)nonylglucosinolate	Glucosinolate	[M − H]⁻ 506.1190	C_17_H_32_NO_10_S_3_^−^	C_17_H_33_NO_10_S_3_	0.7	488.1084 426.1621 96.9595	^61^	-	-	-	-	+	-
77.	4.589	Cyanidin- *O*-deoxyhexosyl-*O*-hexosylpentoside	Anthocyanin	[M + H]^+^ 727.2076	C_32_H_39_O_19_^+^	C_32_H_38_O_19_	1.1	565.1534 433.1121 287.0540			-	-	-	+	-
78.	4.609	Prinsepiol-*O*-hexoside	Lignan	[M − H]⁻ 551.1760	C_26_H_31_O_13_ -	C_26_H_32_O_13_	1.8	389.1223		-	-	-	+	+	-
79.	4.625	Isorhamnetin-*O*-deoxyhexoside-*O*-di-hexoside	Flavonoid	[M + H]^+^ 787.2273	C_34_H_43_O_21_^+^	C_34_H_42_O_21_	2.4	625.1807 463.1213 317.0653		-	-	-	-	+	-
80.	4.643	Kaempferol- *O*-acyl-*O*-di –pentoside	Flavonoid	[M − H]⁻ 725.1912	C₃₂H₃₇O₁₉^−^	C₃₂H₃_8_O₁₉	2.3	725.19112 593.1489 417.0817 285.0396		-	-	-	-	+	-
81.	4.649	Cyanidin- *O*-deoxyhexosyl-*O*-pentoside	Anthocyanin	[M + H]^+^ 565.1555	C_26_H_29_O_14_^+^	C_26_H_28_O_14_	−0.6	433.1118 287.0542		-	-	-	-	+	-
82.	4.658	Delphinidin- *O*-deoxyhexosyl-*O*-pentoside	Anthocyanin	[M + H]^+^ 581.1501	C_26_H_29_O_15_^+^	C_26_H_28_O_15_	0.1	449.1017 303.0493		-	-	-	-	+	-
83.	4.786	Quercetin-*O*-deoxyhexosylihexoside	Flavonoid	[M − H]⁻ 771.1953	C_33_H_39_O_21_^−^	C_33_H_40_O_21_	4.8	301.1190		+	-	-	-	+	-
84.	4.83	Quercetin-*O*-deoxyhexosyl-*O*-hexoside	Flavonoid	[M − H]⁻ 609.08	C_27_H_29_O_16_^−^	C_27_H_30_O_16_	−1.8	463.1785 301.1631		+	+	-	-	+	-
85.	4.94	Kaempferol-*O*-(methoxycaffeoyl)-*O*-dihexoside	Flavonoid	[M − H]⁻ 801.1896	C_37_H_37_O_20_^−^	C_37_H_38_O_20_	−2.0	609.1478 287.1517		-	-	-	-	+	-
86.	4.969	Kaempferol*-O*-deoxyhexosyl-*O*-pentoside	Flavonoid	[M − H]⁻ 563.1418 [M + H]^+^ 565.1553	C_26_H_27_O_14_^−^ C_26_H_29_O_14_^+^	C_26_H_28_O_14_	−2.3 0.7	431.019		-	-	-	-	+	-
87.	5.003	Delphinidin -*O*-deoxyhexoside	Anthocyanin	[M + H]^+^ 449.1070	C_21_H_29_O_14_^+^	C_21_H_28_O_14_	1.2	303.0495	^29^	-	-	-	+	+	-
88.	5.037	Cyanidin-*O*-pentoside	Anthocyanin	[M + H]^+^ 419.097	C_20_H_19_O_10_^+^	C_20_H_18_O_10_	2.0	287.0541		-	-	-	+	+	-
89.	5.09	Isorhamnetin-*O*-rutinoside	Flavonoid	[M − H]⁻ 623.095 [M + H]^+^ 625.1752	C_28_H_31_O_16_^−^ C_28_H_33_O_16_^+^	C_28_H_32_O_16_	1.6 1.5	447.0127		+	-	-	-	+	-
90.	5.138	Unknown acylated flavonoid glycoside	Flavonoid	[M − H]⁻ 739.2076 [M + H]^+^ 741.2272	C_33_H_39_O_19_^−^ C_33_H_39_O_19_^+^	C_33_H_40_O_19_	−1.5	623.1606 563.1396 392.1026 193.0504		-	-	-	-	+	-
91.	5.139	Cyanidin-di-*O*-deoxyhexoside	Anthocyanin	[M + H]^+^ 579.1708	C_27_H_31_O_14_^+^	C_27_H_30_O_14_	5.3	433.1126 287.0550	^31^	-	-	-	+	+	-
92.	5.142	Kaempferol- *O*-hexosyl-*O*-di-deoxyhexoside	Flavonoid	[M − H]⁻ 739.2100 [M + H]^+^ 741.2012	C_33_H_39_O_19_^−^	C_33_H_40_O_19_	3.1	593.1515 431.0960 285.0349		-	-	-	-	+	-
93.	5.176	Quercetin- *O*-tri-deoxyhexoside	Flavonoid	[M − H]⁻ 739.2052	C_33_H_39_O_19_^−^	C_33_H_40_O_19_	3.1	447.0892 301.0348		-	-	-	-	+	-
94.	5.26	Syringic acid-*O*-hexoside	Phenolic acid	[M − H]⁻ 359.0984	C_15_H_19_O_10_^−^	C_15_H_20_O_10_	−0.2	197.0455179.0349		-	-	+	+	+	-
95.	5.34	Kaempferol–O–(pentosyl–(coumaroyl)–pentoside)	Flavonoid	[M − H]⁻ 771.1798	C_36_H_35_O_19_^−^	C_36_H_36_O_19_	−2.1	639.1587 385.1152 315.1820 163.0409 119.0510		+	-	-+	+	-	-
96.	5.379	Isorhamnetin-*O*-deoxyhexosyl-*O*-pentoside	Flavonoid	[M + H]^+^ 595.1650	C_27_H_31_O_15_^+^	C_27_H_30_O_15_	1.2	463.1226 317.0649		-	-	-	+	-	-
97.	5.39	Neoglucobrassicin	Glucosinolate	[M − H]⁻ 477.0646	C_17_H_21_N_2_O_10_S_2_^−^	C_17_H_22_N_2_O_10_S_2_	2.3	446.0445 283.9877 96.9609	^57^	++	+	-	-	-	-
98.	5.42	Quercetin -*O*-glucuronide	Flavonoid	[M − H]⁻ 447.0392	C_21_H1_7_O_13_^−^	C_21_H1_8_O_13_	8.3	175.0392		+	-	-	+	-	-
99.	5.43	Isorhamnetin-*O*-pentosyl-tri-*O*-hexoside	Flavonoid	[M − H]⁻ 933.1791	C_39_H_49_O_26_^−^	C_39_H_50_O_26_	5.4	771.1789 639.1603 477.0645 315.1823		-	-	-	-	+	-
100.	5.620	Quercetin -*O*-hexoside	Flavonoid	[M − H]⁻ 463.0892	C_21_H_19_O_12_^−^	C_21_H_20_O_12_	−2.3	301.0351		+	+	-	-	-	-
101.	5.820	p-Coumaroyl-malic acid	Phenolic acid	[M − H]⁻ 279.0526	C_13_H_11_O_7_^−^	C_13_H_12_O_7_	5.5	133.0154		-	-	-	+	+	-
102.	5.933	Ferulic acid	Phenolic acid	[M − H]⁻ 193.0506	C_10_H_9_O_4_^−^	C_10_H_10_O_4_	0.1	178.0278 134.0377		-	-	-	+	+	-
103.	5.935	Hydroxycoumarin-*O*-hexoside	Coumarin	[M − H]⁻ 339.0662	C_15_H_15_O_9_^−^	C_15_H_16_O_9_	−1.3	177.0184		-	-	+	-	-	-
104.	5.950	Sinapoyl-*O*-malate	Phenolic acid	[M − H]⁻ 339.0741	C_15_H_15_O_9_^−^	C_15_H_16_O_9_^−^	−5.8	223.0618		-	-	-	+	-	-
105.	6.010	Feruloyl-*O*-malate	Phenolic acid	[M − H]⁻ 309.029	C_14_H_13_O_8_^−^	C_14_H_14_O_8_	6.4	193.0501115.0031	^32^	+	+	+	+	-	-
106.	6.050	Ferulic acid derivative	Phenolic acid	[M − H]⁻ 339.0737	C_15_H_15_O_9_^−^	C_15_H_16_O_9_	−5.8	193.0517	^64^	-	-	-	+	+	-
107.	6.070	Unknown	-	[M − H]⁻ 619.064	C_28_H_27_O_16_^−^	C_28_H_28_O_16_	0.1	457.1032		+	-	-	-	-	-
108.	6.183	Isorhamnetin-*O*-(p-coumaroyl)-O-hexoside	Flavonoid	[M − H]⁻ 755.1768	C_36_H_35_O_18_^−^	C_36_H_36_O_18_	8.0	315.1793		-	+	-	-	-	-
109.	6.300	Caffeoyl-*O*-hexoside	Phenolic acid	[M − H]⁻ 341.0915	C_15_H_17_O_9_^−^	C_15_H_18_O_9_	−10.3	180.9824		+	-	+	-	+	+
110.	6.450	Diferuloyl-*O*-malate	Phenolic acid	[M − H]⁻ 505.1362	C_24_H_25_O_12_^−^	C_24_H_26_O_12_	−2.6	389.1277 193.0542		+	-	-	+	+	-
111.	6.620	Quercetin-*O*-deoxyhexoside	Flavonoid	[M − H]⁻ 447.0931	C_21_H_19_O_11_^−^	C_21_H_20_O_11_	0.3	301.0588		+	+	-	-	-	-
112. **-**	6.640	Kaempferol-*O*-hexoside	Flavonoid	[M − H]⁻ 447.0934	C_21_ H_19_ O_11_^−^	C_21_ H_20_ O_11_	−0.2	285.0410		+	+	+	+	+	+
113.	6.920	Rhamnetin –*O*-hexoside	Flavonoid	[M − H]⁻ 477.053	C_22_H_21_O_12_^−^	C_22_H_22_O_12_	1.7			+	+	-	-	+	-
114.	7.202	Kaempferol-*O*-pentoside	Flavonoid	[M − H]⁻ 417.0823	C_20_H_17_O_10_^−^	C_20_H_18_O_10_	0.4	284.0318		-	-	-	-	+	-
115.	7.530	Azelaic acid	Dicarboxylic acid	[M − H]⁻ 187.0969	C_9_H_15_O_4_^−^	C_9_H_16_O_4_	3.8	169.0888 125.0965		+	+	+	-	+	-
116.	7.790	Oxododecanedioic acid	Fatty acid	[M − H]⁻ 243.1232	C_12_H_19_O_5_^−^	C_12_H_20_O_5_	2.7	225.1126 207.1027 181.1233		-	+	+	-	-	-
117.	8.100	Dihydroxy-dioxooctadecanoic acid	Fatty acid	[M − H]⁻ 343.2110	C_18_H_31_O_6_^−^	C_18_H_32_O_6_	4.8	255.1629 229.1431		+	+	-	-	+	-
118.	8.354	Kaempferol-*O*-deoxyhexoside	Flavonoid	[M − H]⁻ 431.0994 [M + H]^+^ 433.1123	C_21_H_19_O_10_^−^ C_21_H_21_O_10_^+^	C_21_H_20_O_10_	−2.4 1.4	285.0403		+	+	+	+	+	+
119.	10.393	Kaempferol	Flavonoid	[M − H]⁻ 285.0402	C_15_H_9_O_6_^−^	C_15_H_10_O_6_	0.8	257.0449 163.0031 125.0238		-	-	+	-	+	-
120.	11.183	Apigenin-*O*-deoxyhexosyl-hexoside	Flavonoid	[M − H]⁻ 577.1608	C_27_H_29_O_14_^−^	C_27_H_30_O_14_	−7.8	269.0452		-	-	-	+	-	-
121.	11.277	Epoxyoctadecatrienoic acid	Fatty acid	[M + H]^+^ 293.2097	C_18_H_29_O_3_^+^	C_18_H_28_O_3_	4.9	275.1997 257.1890 163.1113 97.1011	^63^	+	-	+	+	+	+
122.	11.292	Indol acetic acid	Nitrogenous	[M + H]^+^ 176.0704	C_10_H_9_NO_2_^+^	C_10_H_8_NO_2_	3.3	161.0467 133.0519		+	-	-	-	+	-
123.	11.313	Oxo-phytodienoic acid	fatty acid	[M + H]^+^ 275.003	C_18_H_27_O_2_^+^	C_18_H_26_O_2_	3.0	257.1897 239.1783		+	-	+	+	+	+
124.	11.345	Trihydroxyoctadecadienoic acid	Fatty acid	[M − H]⁻ 327.2172	C_18_H_31_O_5_^−^	C_18_H_32_O_5_	1.6	309.2078 291.1971		+	+	+	+	+	+
125.	11.465	Methoxyspirobrassinin	Glucosinolate	[M − H]⁻ 279.1610	C_12_H_27_N_2_ OS_2_^−^	C_12_H_28_N_2_ OS_2_	−7.3	261.1495 207.1051 97.0652		+	+	+	+	+	+
126.	11.689	Trihydroxyoctadecenoic acid	Fatty acid	[M − H]⁻ 329.2327	C_18_H_33_O_5_^−^	C_18_H_34_O_5_^−^	2	229.1056 211.1254		+	+	+	+	+	+
127.	12.54	Hydroxy-oxooctadecatrienoic acid	Fatty acid	[M − H]⁻ 307.1914 [M + H]^+^ 309.2051	C_18_H_27_O_4_^−^ C_18_H_29_O_4_^+^	C_18_H_28_O_4_	0.3 3.1	289.1532 235.1342		++	+	++	+	+	+
128.	13.039	Gallocatechin-(epi)catechin-(epi)catechin	Flavonoid	[M − H]⁻ 881.4929	C_45_H_37_O_19_^−^	C_45_H_38_O_19_	7.8	593.2638 305.1762	^65^ ^65^	+	-	-	+	+	+
129.	13.29	Octadecenedioic acid	Fatty acid	[M + H]^+^ 311.189	C_27_H_31_O_15_^+^	C_27_H_30_O_15_	3.8	223.2354		+	+	+	+	+	+
130.	13.9	Unknown	Triterpenoid	[M − H]⁻ 721.3655	C_34_H_57_O_16_^−^	C_34_H_58_O_16_	3.7	675.3602 415.1436 397.1358 277.2171 235.0803	^66^	+	+	-	-	-	-
131.	13.952	Octadecanedioic acid	Fatty acid	[M − H]⁻ 313.2375	C_18_H_33_O_4_^−^	C_18_H_34_O_4_	2.9	225.2582		+	+	+	+	+	+
132.	14.44	Hydroxylinolenic acid	Fatty acid	[M − H]⁻ 293.2117	C_18_H_29_O_3_^−^	C_18_H_30_O_3_	1.6	275.1346 235.2431		+	+	+	+	+	+
133.	14.666	Unknown	Steroid	[M + H]^+^ 391.2453	C_23_H_35_O_5_^+^	C_23_H_34_O_5_	6.7	373.2593	^67^	+	+	+	+	+	+
134.	14.735	Unidentified Brassinosteroid	Steroid	[M + H]^+^ 417.2390	C_28_H_33_O_3_^+^	C_28_H_32_O_3_	8.3	399.3590		+	+	-	+	-	-
135.	14.822	Octadecenedioic acid	Fatty acid	[M − H]⁻ 311.2229	C_18_H_31_O_4_^−^	C_18_H_32_O_4_	−0.2	293.2102		+	+	+	+	+	+
136.	14.832	Monolinolenin	Fatty acid	[M + H]^+^ 353.2682	C_21_H_37_O_4_^+^	C_21_H_36_O_4_	1.3	335.2571 261.2208	^68^	+	+	+	+	+	+
137.	15.04	Hydroxylinoleic acid	Fatty acid	[M − H]⁻ 295.2276	C_18_H_31_O_3_^−^	C_18_H_32_O_3_	0.9	277.1036 195.1236		+	+	+	+	+	+
138.	15.509	Lupeol	Triterpene	[M + H]^+^ 425.3753	C_30_H_49_O^+^	C_30_H_48_O	5.9	407.2772	^69^	+	-	-	+	-	+
139.	15.627	Hydroxystigmastenone	Steroid	[M + H]^+^ 429.3706	C_29_H_49_O_2_^+^	C_29_H_48_O_2_	4.9	411.3592 393.3108		+	-	+	-	+	-
140.	15.812	Stearidonic acid	Fatty acid	[M + H]^+^ 277.2155	C_18_H_29_O_2_^+^	C_18_H_28_O_2_	1.5			+	-	+	+	-	+
141.	15.93	Linolenoylglycerol	Fatty acid	[M − H]⁻ 351.2546	C_21_H_35_O_4_^−^	C_21_H_36_O_4_	−0.7	333.249		+	+	+	+	+	+
142.	15.963	SQDG (16:1/16:0)	Sulfolipid	[M − H]⁻ 791.4956	C_41_H_75_O_12_S^−^	C_41_H_76_O_12_S	3.6	537.3298 381.3379 249.1867	^70^ ^36^	+	+	-	-	-	-
143.	16.01	SQDG (18:3/16:0)	Sulfolipid	[M − H]⁻ 817.424	C_43_H_77_O_12_S^−^	C_43_H_78_O_12_S	1.4	561.3261 539.3286 297.2425		+	+	-	+	-	-
144.	16.11	Unknown SQDG(18:1/16:1)	Sulfolipid	[M − H]⁻ 819.436	C_43_H_79_O_12_S^−^	C_43_H_78_O_12_S	7.8	566.3446 540.3283 279.2322		+	+	-	+	+	+
145.	16.337	Palmitate-*O*-hexoside	Fattyacid	[M − H]⁻ 417.2856	C_22_H_41_O_7_^−^	C_22_H_42_O_7_	0.5	255.2320		+	+	-	+	+	+
146.	16.82	Linolenic acid	Fatty acid	[M − H]⁻ 277.187	C_18_H_29_O_2_^−^	C_18_H_30_O_2_	0.4	215.2163		+	+	+	+	+	+
147.	17.000	Hydroxyl palmitic acid	Fatty acid	[M − H]⁻ 271.2279	C_16_H_31_O_3_^−^	C_16_H_32_O_3_	−0.0	225.2220		+	+	+	+	+	+
148.	17.43	Linoleic acid	Fatty acid	[M − H]⁻ 279.2326	C_18_H_31_O_2_^−^	C_18_H_32_O_2_	1.4	261.2218 235.2425 171.1385		+	+	+	+	+	+
149.	17.982	Stigmasterone	Steroid	[M + H]^+^ 411.3620	C_29_H_47_O^+^	C_29_H_46_O	0.4	393.3621 313.3610	^71^	+	-	-	-	-	+

#### Phenolic acids and their glycosides

The negative ionization mode led to the detection of 16 phenolic acids either free or conjugated with sugars and/or organic acids. The major phenolic acids detected in cruciferous leafy vegetables included quinic acid (peak 9) [(M-H)^−^ *m/z* 191.0566 (C_7_H_11_O_6_)^−^], cinnamic acid (peak 28) [(M-H)^−^ *m/z* 147.0456 (C_9_H_7_O_2_)^−^], and ferulic acid (peak 102) [(M-H)^−^*m/z* 193.0506 (C_10_H_9_O_4_)^−^]. Phenolic acid glycosides were also detected in gentisic acid-*O*-hexoside (peak 32) [(M-H)^−^ *m/z* 315.0737 (C_13_H_15_O_9_)^−^], vanilllic acid-*O*-hexoside (peak 33) [(M-H)^−^ *m/z* 329.0884 (C_14_H_17_O_9_)^−^], feruloyl-*O*-hexoside (peak 61) [(M-H)^−^*m/z* 355.1042 (C_16_H_19_O_9_)^−^], and caffeoyl-*O*-hexoside (peak 109) [(M-H)^−^ *m/z* 341.0915 (C_15_H_17_O_9_)^−^] with the main fragment ion peaks at *m/z* 153.0194, *m/z* 167.0351, *m/z* 193.0520, *m/z* 223.0560 and 180.9824, respectively, corresponding to the deprotonated phenolic acid aglycone. Sinapic acid and sinapoyl esters are commonly reported in Brassicaceae family^21,22^, and to account for its leafy taste. Several sinapic acid glycosidic conjugates were annotated including sinapoyl-*O*-hexoside (peak 69)[(M-H)^−^*m/z* 385.1133 (C_17_H_21_O_10_)^−^] with fragment ion at *m/z* 223.0560 [M-H-162]^−^ of deprotonated sinapic acid,, and peak 104 for sinapoyl-*O*- malate[(M-H)^−^ *m/z* 339.0741 (C_15_H_15_O_9_)^−^] that was detected only in turnip with fragment ion peak at *m/z* 223.0618 of deprotonated sinapic acid. Malate esters were likewise detected in phenolic acids including *p-*coumaroyl-malic acid (peak 101) [(M-H)^−^ *m/*z 279.0526(C_13_H_11_O_7_^−^)^−^] found exclusively in turnip, and feruloyl-*O*-malate (peak 105) [(M-H)^−^ *m/z* 339.0741 (C_15_H_15_O_9_)^−^], based on the main loss of 116 amu corresponding to malic acid moiety. The examined 6 Brassicaceae samples showed qualitative differences in phenolic acid esters, in which feruloyl malate [(M-H)^−^ *m/z* 339.0741 (C_15_H_15_O_9_)^−^] and coumaroyl malate [(M-H)^−^ *m/z* 339.0741 (C_15_H_15_O_9_)^−^] were most prominent in turnip and radish.

Organic acids such as gluconic acid (peak 2) [(M-H)^−^*m/z* 195.0507 (C_6_H_11_O_7_)^−^], malic acid (peak 10) [(M-H)^−^ *m/z* 133.0331 (C_4_H_5_O_5_)^−^] and citric acid (peak 13) [(M-H)^−^ *m/z* 191.0186 (C_6_H_7_O_7_)^−^] were also detected, in which gluconic acid was the most abundant acid in 3 brassica taxa including the non-edible leaves, while present at low levels in turnip, radish, and watercress. Organic acids, including malic and citric acids, play a pivotal role in food safety acting as acidulant preservative^23^.

#### Flavonoid glycosides

A total of 42 flavonoids were annotated, of which 28 were flavonoid glycosides versus 14 acylated flavonoid glycosides. The identified flavonoids were mainly glycosylated derivatives of three flavonols viz., kaempferol, quercetin, and isorhamnetin, while only one apigenin conjugate was detected in turnip. The sugar moieties found in cruciferous vegetables typically occur as mono-, di-, tri-, tetra-, and pentaglycosides^24^, they are also commonly found acylated by different hydroxycinnamic acids^25^, to further improve their absorption and cellular target interaction with myriad of health benefits including antimicrobial, antiparasitic, anti-inflammatory, anti-nociceptive, analgesic, and anti-complementary^26^. In MS/MS analysis, the nature of sugars and acyl groups could be revealed from elimination of the sugar residues, that are 162 amu for hexose (C_6_H_10_O_5_), 146 amu for deoxyhexose (C_6_H_10_O_4_) and 132 for pentose (C_5_H_8_O_4_),while acyl groups showed the losses of 176 amu for feruloyl, 162 amu for caffeoyl, 146 amu for *p*-coumaroyl, 206 for sinapoyl, 192 amu for methoxycaffeoyl and 116 for malic acid moieties. Analysis of crucifers revealed that the highly glycosylated flavonoids, including tetra- and penta-hexosides are more characteristic of the four *Brassica* taxa, albeit not detected in radish and watercress. In detail, highly glycosylated non-acylated flavonoids were found in negative mode as tetra-glycosides of quercetin, kaempferol, and isorhamnetin in peaks 40 with *m/z* 933.2563, peak 41 with *m/z* 933.2478, peak 99 with *m/z* 933.1791,and peak 66 with *m/z* 887.2451(C_38_H_47_O_24_). MS^2^ Fragment ions further distinguished between structural isomers in peaks 39, 45 and 99. For example in case of peak 40, fragment ion at *m/z* 771 [M-H-162 (C_6_H_10_O_5_)]^−^, *m/z* 609 [M-H-162 (C_6_H_10_O_5_)−162 (C_6_H_10_O_5_)]^−^, *m/z* 489 [M-H-162 (C_6_H_10_O_5_)−162 (C_6_H_10_O_5_)−120]^−^ and aglycon fragment ion of kaempferol at *m/z*285 [M-H-162 (C_6_H_10_O_5_)−162 (C_6_H_10_O_5_)−324 (C_12_H_20_O_10_)]^−^ was assigned as kaempferol-*O*- dihexoside–di-*O-*hexoside. While MS^2^ fragment ions for peak 41 at *m/z* 771 [M-H-162 (C_6_H_10_O_5_)]^−^, *m/z* 447 [M-H-162 (C_6_H_10_O_5_)−324 (C_12_H_20_O_10_)]^−^ and *m/z* 301 [M-H-162 (C_6_H_10_O_5_)−324 (C_12_H_20_O_10_)−146 (C_6_H_10_O_4_)]^−^ was identified as quercetin-*O*-dihexoside-*O*-hexosyl-deoxyhexoside. Peak 99 MS^2^ fragment ions at *m/z* 771 [M-H-162 (C_6_H_10_O_5_)]^−^, *m/z* 639 [M-H-162 (C_6_H_10_O_5_)−132 (C_5_H_8_O_4_)]^−^, *m/z* 477 [M-H-162 (C_6_H_10_O_5_)−132 (C_5_H_8_O_4_)−162 (C_6_H_10_O_5_)]^−^ and *m/z* 315 relative to isorhamnetin post loss of another hexose moiety was identified as isorhamnetin-*O*-pentosyl-tri-*O*-hexoside (Suppl. **Fig. ****S1**).

Peak 66 showed MS^2^ fragment ions at *m/z*755[M-H-132 (C_5_H_8_O_4_)]^−^due to loss of pentose moiety, *m/z*593[M-H-132 (C_5_H_8_O_4_)−162 (C_6_H_10_O_5_)]^−^, and *m/z* 431[M-H-132 (C_5_H_8_O_4_)−162 (C_6_H_10_O_5_)−162 (C_6_H_10_O_5_)]^−^assigned it as kaempferol-*O*-pentosyldeoxyhexoside-di-*O*-hexoside.

A total of 14 flavonol glycosides were acylated with hydroxycinnamic acids i.e., ferulic, caffeic, sinapic, and coumaric acid. They were detected most prominent in the 4 cruciferous viz., cabbage, broccoli, cauliflower, and turnip. For example, peak 36 [(M-H)^−^ *m/z* 1095.2897 (C_48_H_55_O_29_^−^)^−^] showed MS^2^ fragment ions at *m/z* 933.2426 [M-H-162 (C_9_H_6_O_3_)]^−^ due to the loss of caffeic acid moiety, *m/z* 787.1872 [M-H-162 (C_9_H_6_O_3_)- 146 (C_6_H_8_O_4_)]^−^ relative to the loss of deoxyhexose sugar and *m/z* 463.0463 [M-H-162 (C_9_H_6_O_3_)- 146 (C_6_H_8_O_4_)−324 (C_12_H_20_O_10_)]^−^ due to the successive sugar loss ultimately yielding aglycon fragment ion at *m/z*301 annotated as quercetin-*O*-caffeoyldeoxyhexosyl–*O*-tri-hexoside (Suppl. **Fig. S2**). Peak 80 [(M-H)^−^ *m/z* 725.1912 (C_32_H_37_O_19_-)^−^] showed fragment ions at *m/z* 593.1489 [M-H-132 (C_5_H_8_O_4_)]^−^ due to the loss of pentose moiety, *m/z* 417.0817 [M-H-132 (C_5_H_8_O_4_)−176 (C_10_H_8_O_3_)]^−^ for the loss of acyl and pentose moieties, identified as kaempferol- *O*-acyl-*O*-di-pentoside (Suppl. **Fig. S3**). A number of identified flavonoids were detected only on radish this include 3 non-acylated flavonoid glycosides as in peak 75 [(M-H)^−^ *m/z* 725.1937(C_32_H_27_O_19_^−^)^−^], peak 92 [(M-H)^−^ *m/z* 739.2100 (C_33_H_39_O_19_^−^)^−^], and peak 93 [(M-H)^−^ *m/z* 739.2052 (C_33_H_39_O_19_^−^)^−^], in addition to 3 acylated flavonoid glycosides as in peaks 67 [(M-H)^−^ *m/z* 741.1876 (C_35_H_33_O_18_^−^)^−^], peak 80 [(M-H)^−^ *m/z* 725.1912 (C₃₂H₃₇O₁₉-)^−^], peak 90 [(M-H)^−^ *m/z* 739.2076 (C_33_H_39_O_19_^−^)^−^] Thus radish may be considered as a prospective extract with potential effects such as antimicrobial, antiparasitic, anti-inflammatory, and analgesic effects^27^.

A proanthocyanidin was identified in peak 128 [(M-H)^−^ *m/z* 881.4929(C_45_H_37_O_19_^−^)^−^] with fragment ions at *m/z* 593 [M-H-288]^−^ and *m/z*305 [M-H-288-288]^−^ due to successive losses of catechin units with quinone methide (QM) fragmentation and appearance of gallocatechin fragment ion peak at *m/z* 305 identified as gallocatechin-(epi)catechin-(epi)catechin detected in all extracts except cauliflower and cabbage^28^ (Suppl. **Fig. S8)**.

#### Anthocyanins

Anthocyanins are widely distributed as colored pigments in different fruits and vegetables^29^. In crucifers, anthocyanins are highly conjugated with different sugars and/or acyl groups to improve their stability^30^. Due to their existence in a positively charged cationic form, they are more sensitive to be detected in positive ionization mode for such flavonoid subclass^29^ and confirmed in this study. Nine anthocyanins were detected belonging to either cyanidin or delphinidin aglycones. For example, peak 39 [(M + H)^+^ *m/z* 935.2672 (C_39_H_51_O_26_^+^)^+^] showed MS/MS ions at *m/z* 773 [M + H-162]^+^, *m/z* 611[M + H-2 × 162 amu]^+^, and finally *m/z* 287 for its aglycone post losses of hexose moieties assigned as cyanidin-*O*-tetra-hexoside-identified in broccoli and cabbage (Suppl. **Fig. S7**). Peak 62 [(M + H)^+^ *m/z* 773.2213 (C_33_H_41_O_21_^+^)^+^] is a delphinidin derivative showed MS/MS ions at *m/z* 611 [M + H-162]^+^,*m/z*449[M + H-2 × 162 amu]^+^, and finally *m/z*303 for delphinidin aglycone post losses of deoxyhexose moiety identified as delphinidin-*O*-deoxyhexoside-di-*O*-hexoside detected in radish and watercress. Among the examined crucifers, anthocyanins were detected in radish followed by turnip, in contrast no anthocyanins were detected in cauliflower. A thorough examination for radish and turnip leaves will be of interest owing to the anti-mutation, liver protection, and inhibiting the metastasis of tumor cells properties of such metabolites^31^.

#### Glucosinolates (GLS)

**Fig. 3:**
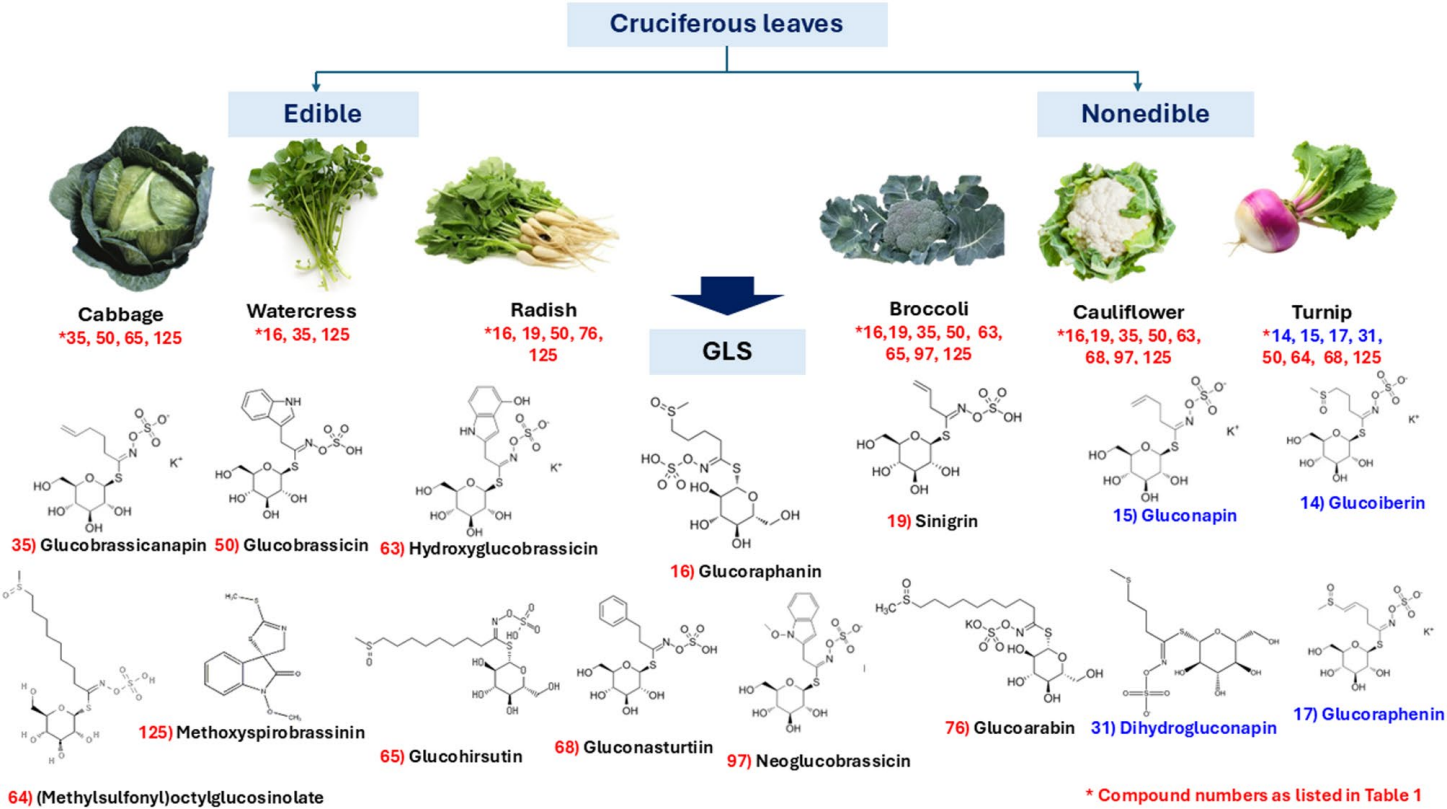
Identified glucosinolates in edible and nonedible cruciferous leaves, the compound in blue color were identified as markers in turnip only.

GLS were detected in the negative ion mode due to the sulfate moiety showing characteristic fragment ions at *m/z* 275, 259, 241, 195, and 97^32^. Fragment ion at *m/z* 259 corresponded to sulfated hexose moiety, while *m/z* 195 was attributed to the thio-glucose moiety. A total of 15 GLS were identified to be more prevalent in non-edible crucifers than edible ones (Fig. 3). This includes peak 14 [(M-H)^−^ *m/z* 422.0228(C_11_H_19_NO_10_S_3_^−^)^−^] identified as glucoiberin and detected in cabbage and turnip only. Characteristic MS^2^ fragment ions at *m/z* 358.0237 [M-H-64 (CH_3_SO)]^−^ indicative of a loss of methyl sulfoxide, *m/z* 195.0123 of thio-glucose residue and *m/z* 96.6577 of the sulfate moiety led to its assignment as glucoiberin^33^ (Suppl. **Fig. S4**), first time to be reported in turnip. Another GLS found only in turnip assigned as gluconapinin peak 15 [(M-H)^−^ *m/z* 372.0422 (C_11_H_18_NO_9_S_2_^−^)^−^] with MS^2^ product ions at *m/z* 292.087 [M-H-80 (SO_3_)]^−^ due to the neutral loss of 80 amu (SO3), and *m/z* 195.057 of thio-glucose moiety (Suppl. **Fig. S5**), following other previously reported studies^34^. Peak16 [(M-H)^−^ *m/z* 436.0426 (C_12_H_22_NO_10_S_3_^−^)^−^] is a prominent GLS identified in broccoli, cauliflower, watercress and radish, exhibiting the main fragment ion peak at *m/z* 195.0421 of thio-glucose residue and identified as glucoraphanin previously reported in *Brassica* species^35^ (Suppl. **Fig. S6**). Several GLS were detected exclusively in radish and warranting for future studies on vegetables targeting its GLS profile including peak 17 [(M-H)^−^ *m/z* 434.0255 (C_12_H_20_NO_10_S_3_^−^)^−^], peak 64 [(M-H)^−^ *m/z* 508.0958 (C_16_H_30_N_2_O_11_S_3_^−^)^−^], and peak 76[(M-H)^−^ *m/z* 506.1129 (C_17_H_32_NO_10_S_3_^−^)^−^] identified as glucoraphenin, (methylsulfonyl)octylglucosinolate, and glucoarabin (methylsulfinyl)nonylglucosinolate. These are aliphatic GLS detected previously in cabbage^18^ and first time reported in radish. In contrast to aliphatic GLS that showed diversity in composition among examined crucifers, indole GLS were detected in all examined crucifers. A major indole-derived GLS detected in all examined Brassicaceae, though most prominent in radish was methoxyspirobrassin in peak 125 [M-H)^−^ *m/z* 279.1610(C_12_H_27_N_2_ OS_2_^−^)^−^]. Although some of the identified GLS were first reported in radish and turnip, non-edible broccoli and cauliflower showed to have more identified GLS. This raises interest in the valorization of such by-products for further analysis. Although certain GLS were newly reported in radish and turnip, non-edible species such as broccoli and cauliflower exhibited higher GLS diversity and abundance, suggesting potential valorization of these agricultural by-products as rich sources of bioactive GLS. This observation aligns with growing interest in the recovery of functional compounds from cruciferous waste streams for nutraceutical and pharmaceutical applications.

#### Fatty acids and fatty acyl esters

A total of 20 fatty acids were detected in examined crucifers, exhibiting relatively minor variationamong examined taxa compared to secondary metabolites such as GLS and flavonoids. Major prominent peaks identified in all samples included peaks 124, 127, 137, and 130 of which several are newly reported in crucifers belonging to hydroxylated fatty acids. Peak 124 [M-H)^−^ *m/z* 327.2721 (C_18_H_31_O_5_^−^)^−^] showed fragment ion peaks at *m/z* 309.2078 [M-H-18]^−^ and *m/z* 291.1971 [M-H-18-18]^−^ due to successive losses of water molecules, identified as trihydroxyoctadecadienoic acid most prominent in broccoli, radish, and turnip. Peak 127 [(M-H)^−^ *m/z* 307 (C_18_H_27_O_4_^−^)^−^] showed fragment ion peaks at *m/z* 289.1532[M-H-18]^−^and *m/z* 235.1342 [M-H-18-18-18]^−^ due to successive losses of water identified as hydroxy-oxo-octadecatrienoic acid being most prominent in broccoli and cabbage. Likewise, peak 137 [M-H)^−^ *m/z* 295.2276 (C_18_H_31_O_3_^−^)^−^] with MS^2^ fragments at *m/z* 277.1036 [M-H-18]^−^ due to loss of water and *m/z* 195 corresponding to OH-CH (CH_2_)_8_COO^−^ fragment identified as hydroxylinoleic acid. Two A glycosylated oxylipid peak 145 [M-H)^−^ *m/z* 417.2856 (C_22_H_41_O_7_^−^)]^−^ are detected in all examined cruciferous leaves except for cabbage and is first time to be reported in Brassicaceae. Identification was based on main fragment ion corresponding to the loss of hexose moiety with MS^2^ fragment at *m/z* 255.232 as in case of peak 130 [M-H)^−^ *m/z* 721 (C_34_H_57_O_16_^−^)^−^. Because fatty acids and their derivatives contribute not only to the nutritional lipid profile of cruciferous vegetables but also to their bioactivity and health-promoting effects, their identification provides an important link between metabolomic profiling and potential functional food applications.

#### Sulfolipids

Sulfoquinovosyl monoacylglycerols are glycolipids (SQMGs) composed of a diacylglycerol esterified with fatty acids and a sulfoquinovose moiety^36^. Three sulfolipids were identified in extracts that are first time to be reported in Brassica showing improved detection in negative mode and suggestive the sulphur is incorporated in lypophilic metabolites in crucifers. MS/MS spectrum of peak 142 [M-H)^−^ *m/z* 791.4956 (C_41_H_75_O_12_S^−^)^−^ showed fragment ions at *m/z* 537 corresponds to loss of (C16:0) fatty acid and *m/z* 249 corresponds to the deprotonated sulfoquinovose identified as SQDG (16:1/16:0) and was detected only in non-edible broccoli and cauliflower. Indeed, broccoli, cauliflower and turnip were the most rich in SQMGs exemplified by SQDG (18:3/16:0) in peak 143[(M-H)- *m/z* 817.424 (C_43_H_77_O_12_S^−^)^−^ showing fragment ions at *m/z* 539, *m/z* 561corresponding to [M–RCOOH]^–^ where RCOOH are the C18:3 and C16:0 fatty acids, respectively, and a fragment ion peak at *m/z* 297.2425 [539–243 (C_6_H_11_O_8_S)]-, due to the loss of sulfoquinovosyl anion (Suppl. **Fig. S9**). In contrast, peak 144 [(M-H)- *m/z* 819.436 (C_43_H_79_O_12_S^−^) showed fragment ions at *m/z* 566.3446, m/z 540.3283 corresponding to [M–RCOOH-H]^–^ where RCOOH are the C18:1 and C16:1 fatty acid, respectively, identified as SQDG (18:1/16:1) was detected in all most extracts. Within examined extracts, broccoli and cauliflower appeared most rich in sulfolipids, and yet to be tested for their role in health effects or to be targeted for isolation from these sources^37^.

#### Nitrogenous compounds

A total of 6 nitrogenous compounds were detected exclusively in cauliflower and radish including peak 56 [(M-H)^−^ *m/z* 452.1662 (C_14_H_30_NO_15_^−^)^−^] with MS^2^ fragments at *m/z* 362, 184and179, peak 58 [(M-H)^−^ *m/z* 372.1271 (C_16_H_22_NO_9_^−^)^−^] and peak 73 [(M-H)^−^ *m/z* 210.0787 (C_10_H_12_NO_4_^−^)^−^] with 162 amu mass differences, and annotated as methoxytyrosin-*O*-hexoside and methoxytyrosin, respectively, posing cauliflower and radish as potential sources of these amino acid conjugates. Although the presence of methoxylated derivatives of tyrosine and related amino acids is previously reported in Brassica^38^, methoxytyrosin-*O*-hexoside and methoxytyrosin are first time to be identified in cauliflower and radish.

### Multivariate data analysis of cruciferous leaves UPLC-MS/MS metabolite dataset

Unsupervised principal component analysis (PCA) model was employed to assess metabolite heterogeneity among examined crucifer leaves. Further, supervised orthogonal projection to latent structures discriminant analysis (OPLS-DA) was employed for classification between edible and non-edible cruciferous, alongside identification of markers.

#### Unsupervised PCA analysis of crucifer leaves

**Fig. 4:**
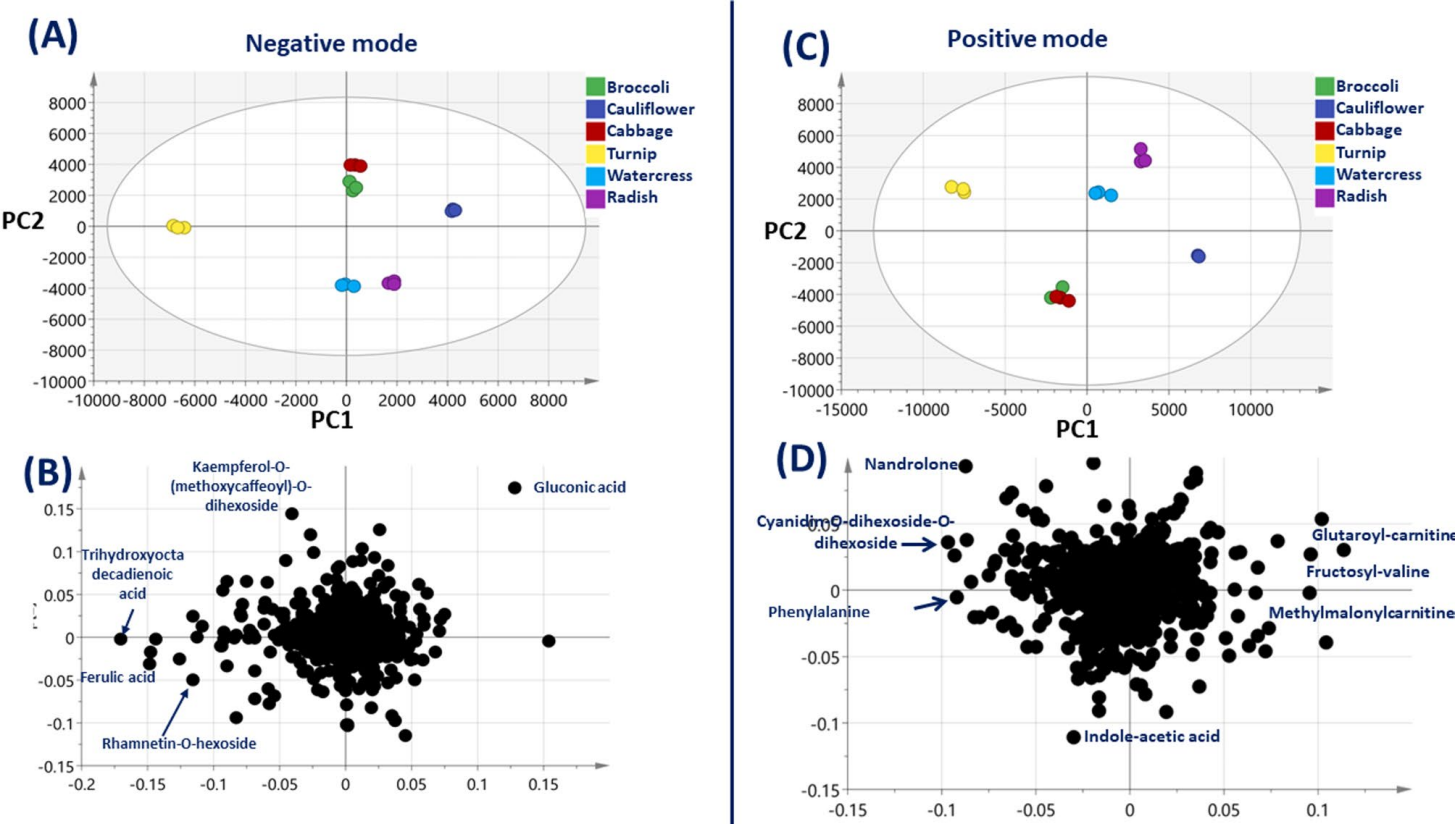
Unsupervised multivariate data analyses of the studied cruciferous leaves derived from modeling secondary metabolites dataset analysed via UHPLC-HRMS/MS. (**A**) PCA score plot of positive mode with PC1 vs. PC2. (**B**)Loading plot for positive mode providing variant peaks and their assignments. (**C**) PCA score plot of negative mode with PC1 vs. PC2. (**D**)Loading plot for negative mode providing variant peaks and their assignments.

A PCA model (Fig. 4) was performed for both positive and negative metabolite datasets, resulting in two independent PCs with R^2^ = 0.799 and Q^2^ = 0.607, and R^2^ = 0.717 and Q^2^ = 0.499, respectively. The PCA score plot of the negative mode dataset (Fig. 4A) revealed clear differentiation of turnip on the left side of PC1, indicating its distinct composition compared to other cruciferous situated in five separate clusters consisting of radish, watercress, cabbage, broccoli, and cauliflower along PC2. Metabolites that influenced segregation were revealed from the corresponding loading plot (Fig. 4B) indicating that some polyphenols such as ferulic acid derivatives (peak106), rhamnetin-*O*-hexoside (peak113), as well as tri-hydroxyoctadecadienoic acid (peak124) were enriched in turnip, turnip, and posing as a rich source of polyphenols in crucifers, yet to be tested using proximate assays for phenolics. In contrast, the presence of gluconic acid accounted for cabbage, broccoli, and cauliflower separate clustering on the positive side of PC1. Gluconic acid was detected at relatively much higher levels in these crucifers compared to turnip, though of less taxonomical value as primary metabolite.

Similarly, PCA score plot for positive mode (Fig. 4C) confirmed the distinct turnip composition, being positioned far to the left of PC1. The close segregation of broccoli and cabbage on the positive left side implied similar metabolite composition and suggests that nonedible broccoli leaves share similarities with edible cabbage. Analysis of the corresponding loading plot (Fig. 4D) revealed richness in cyanidin-O-dihexoside-O-dihexoside and phenylalanine-*O*-hexoside in turnip. In contrast, the segregation of broccoli and cabbage was influenced by their richness in indole-acetic acid (peak 122). Additionally, loading plot revealed acyl carnitines exemplified by glutaroyl-carnitine (peak 21), fructosyl-valine (peak18), and methylmalonylcarnitine peak 12 in radish, cauliflower, and watercress posing them as potential source of acylcarnitines, especially considering their anti-obesity effects as they play a pivotal role in energy production and metabolism of fatty acids^39^.

#### Supervised OPLS-DA modelling of crucifer leaves

Compared to unsupervised PCA analysis, OPLS-DA allows for the maximum separation between groups and can identify key metabolites that contribute to the differentiation in a supervised pairwise manner. The first OPLS-DA model was developed to confirm turnip marker metabolites that showed distinct separation from other cruciferous vegetables in PCA analysis (Fig. 4). OPLS-DA Models were constructed for positive (**Fig. S10A**) and negative (**Fig. S10C**) ion mode dataset revealed distinct separation of turnip from other crucifers along the predictive component. The models showed high predictive capability with Q^2^ = 0.98, R^2^Y = 0.99 and Q^2^ = 0.99, R^2^Y = 0.99, for positive and negative datasets, respectively. Moreover, permutation tests confirmed the robustness of the models with negative Q^2^ intercept values and further validated the significance of the observed separation between turnip and other cruciferous vegetables. CV-ANOVA tests with p values less than 0.05 indicated model significance, further supporting the observed separation between turnip and other cruciferous vegetables. Metabolites that influence such patterns can be verified from the respective loading S-plots of negative (**Fig. S10B**) and positive (**Fig. S10D**) datasets. Interestingly, mostly polyphenols viz. ferulic acid, and rhamnetin–O-hexoside and cyanidin-O-dihexoside-O-dihexoside, that appeared to be significantly higher in turnip compared to other crucifer vegetables, and in agreement with PCA results. In addition, turnip was also enriched in primary metabolites viz. tri-hydroxyoctadecadienoic acid and phenylalanine-*O*-hexoside, which further contribute to its unique metabolite composition compared to other cruciferous vegetables.

The second OPLS-DA model aimed to analyze metabolite composition of cabbage as an edible cruciferous vegetable and compared with other vegetables. The OPLS-DA analysis (**Fig. S11**) revealed clear differentiation of cabbage and other cruciferous vegetables, indicating that cabbage has a distinct flavonoid composition. Of these flavonoids, quercetin-O-deoxyhexosyl-O-hexoside, quercetin-dihexoside-*O*-deoxyhexoside were enriched in cabbage compared to other crucifers/quercetin has potential antioxidant, anti-inflammatory, in addition to anti-cancer properties^40^. Other metabolites belonging to fatty acids i.e. stearidonic acid and hydroxylinolenic acid were found enriched in cabbage compared to other cruciferous vegetables. Another OPLS-DA **(Fig. S12**) was employed to model broccoli against other edible crucifers, showing clear separation of broccoli along the predictive component. Key markers for broccoli included quinic acid, neoglucobrassicin, and indole acetic acid.

A final OPLS-DA comparison was adopted to model cabbage as an example of edible cruciferous vegetable in one class against broccoli as non-edible crucifer in another class **(Fig. S13)**. Clear separation was observed attributed to quercetin-*O*-deoxyhexosyl-*O*-hexoside, quercetin-dihexoside-*O*-deoxyhexoside, and gluconic acid richness in cabbage.

### Marker metabolites quantification and statistical analysis

The one-way ANOVA results revealed highly significant differences (FDR-adjusted *p* < 0.001) among cruciferous species for all identified marker metabolites (Table 2), confirming strong metabolic differentiation within the dataset. The abundance of top 10 significant metabolites based on peak area was listed in **Table ****S1**. Phenolic acids and flavonoid glycosides are the major contributors to interspecies metabolic variability within cruciferous vegetables. The boxplots of marker metabolites detected in the negative ionization mode (**Figure S14**) clearly demonstrate distinct species-dependent variations in metabolite accumulation among cruciferous vegetables. Turnip and watercress were enriched in quercetin-, rhamnetin-, and kaempferol-glycosides, while broccoli and cabbage exhibited higher levels of organic acids such as gluconic and malic acids. Lipid-derived metabolites such as trihydroxyoctadecadienoic acid and palmitoleic-linolenic-*O*-hexoside were more prominent in broccoli and cauliflower.

**Table 2:** Statistical significance of marker metabolites identified in cruciferous vegetables detected in UPLC-MS/MS analysis in negative mode.

Metabolite	F-statistic	*p*-value	FDR adj. *p*-value	Effect Size (η²)	Significance
Quercetin-O-deoxyhexosyl-O-hexoside	3803.69	< 0.001	< 0.001	0.999	***
Ferulic acid	2263.14	< 0.001	< 0.001	0.999	***
Trihydroxyoctadecadienoic acid	1715.99	< 0.001	< 0.001	0.999	***
Rhamnetin –O-hexoside	1421.77	< 0.001	< 0.001	0.998	***
Feruloyl malate	1389.33	< 0.001	< 0.001	0.998	***
Quercetin hexoside deoxyhexoside-O- hexoside	1205.96	< 0.001	< 0.001	0.998	***
Isorhamnetin-O-hexosyl pentoside-O-hexoside	802.46	< 0.001	< 0.001	0.997	***
Kaempferol-O-hexosylhexoside	569.38	< 0.001	< 0.001	0.996	***
Isorhamnetin-O-(coumaroyl)-hexoside	536.39	< 0.001	< 0.001	0.996	***
Gluconic acid	522.56	< 0.001	< 0.001	0.995	***
Gluconapin	381.36	< 0.001	< 0.001	0.994	***
p-Coumaric acid	207.03	< 0.001	< 0.001	0.989	***
Malic acid	186.54	< 0.001	< 0.001	0.987	***
Palmitoleic-linolenic-O- hexoside	65.61	< 0.001	< 0.001	0.965	***
Hydroxylinolenic acid	13.02	< 0.001	< 0.001	0.844	***

*ANOVA results for metabolites with FDR-adjusted p < 0.05*. *** p < 0.001, ** p < 0.01, * p < 0.05. *η² = eta-squared (effect size)*.*n = 3 biological replicates per species*.

 In the positive ionization mode (Table 3), all detected marker metabolites displayed highly significant interspecies differences (*p* < 0.001, FDR-adjusted *p* < 0.001), with large effect sizes (η^2^ > 0.92), indicating strong metabolic discrimination among cruciferous vegetables. The abundance of top 10 significant metabolites based on peak areas was listed in **Table S2**. The most discriminative metabolites were the anthocyanin derivatives delphinidin-*O*-deoxyhexoside and cyanidin-*O*-tetra-hexoside. Amino acid derivatives such as fructosyl-valine, hexose phenylalanine, and indole-acetic acid also showed strong species-dependent differences (η^2^ = 0.985–0.990). Lipid-related compounds, including monolinolenin and stearidonic acid, exhibited slightly lower but still substantial η^2^ values (0.974–0.981). Boxplots of the metabolites detected in the positive ionization mode (**Figure S15**) reveal clear interspecies differences in metabolite abundance, further emphasizing the metabolic diversity within cruciferous vegetables.

**Table 3:** Statistical significance of marker metabolites identified in cruciferous vegetables detected in UPLC-MS/MS analysis in positive mode.

Metabolite	F-statistic	*p*-value	FDR adj. *p*-value	Effect Size (η²)	Significance
Delphinidin -O-deoxyhexoside	585.30	< 0.001	< 0.001	0.996	***
Cyanidin-O-tetra-hexoside	500.48	< 0.001	< 0.001	0.995	***
Methylmalonylcarnitine	413.59	< 0.001	< 0.001	0.994	***
Oxo-phytodienoic acid	338.71	< 0.001	< 0.001	0.993	***
Fructosyl-valine	243.01	< 0.001	< 0.001	0.990	***
Delphinidin-O-deoxyhexoside-di-O-hexoside	170.20	< 0.001	< 0.001	0.986	***
Hexose phenylalanine	159.39	< 0.001	< 0.001	0.985	***
Indole-acetic acid	157.89	< 0.001	< 0.001	0.985	***
Monolinolenin	121.73	< 0.001	< 0.001	0.981	***
Stearidonic acid	91.55	< 0.001	< 0.001	0.974	***
Glutaroyl-carnitine	29.89	< 0.001	< 0.001	0.926	***

*ANOVA results for metabolites with FDR-adjusted p < 0.05*. *** p < 0.001, ** p < 0.01, * p < 0.05. *η² = eta-squared (effect size)*.*n = 3 biological replicates per species*.

## Conclusion

Secondary metabolites heterogeneity in 6 cruciferous leafy vegetables in the context of being edible and none-edible is introduced herein through UPLC-MS/MS-based metabolomics coupled with multivariate data analyses. UPLC-MS/MS analysis led to the detection of 149 peaks, including mostly phenolic acids, flavonoids, fatty acids/acyl esters, steroids, anthocyanins, and glucosinolates. Several identified metabolites are newly reported in crucifers belonging to hydroxylated fatty acids, oxylipid, sulfolipids, and tyrosine derivatives, besides a number of detected GLS for the first time in turnip include glucoiberin, and radish including (glucoraphenin, (methylsulfonyl)octylglucosinolate, and glucoarabin (methylsulfinyl)nonylglucosinolate)). Nonedible broccoli and cauliflower showed exclusively a number of identified compounds belonging to GLS, flavonoids and sulfolipids, posing them to be tested for their role in health effects or to be targeted for isolation from these sources. Turnip is shown to have a more characteristic profile based on the identified metabolites belonging to phenolic acids, sinapoyl derivatives, and anthocyanins. Multivariate data analysis revealed that turnip was the most distinct among all examined leaves. ferulic acid derivatives, and rhamnetin-*O*-hexoside, alongside tri-hydroxyoctadecadienoic acid were key metabolites helped in discrimination of turnip from other samples. Quercetin-*O*-deoxyhexosyl-*O*-hexoside, quercetin hexoside deoxyhexoside-*O*-hexoside, gluconic acid, hydroxylinolenic acid, and palmitoleic-linolenic-*O*-hexoside were enriched in cabbage than other cruciferous leaves. Moreover, quinic acid, and neoglucobrassicin were enriched in broccoli as nonedible cruciferous vegetable while, quercetin-O-deoxyhexosyl-O-hexoside, quercetin-dihexoside-*O*-deoxyhexoside, and gluconic acid were enriched in edible cabbage leaves. A limitation of this study is the relatively small sample size, with only three biological replicates analyzed per variety. However, the consistent clustering and clear separation observed in the multivariate analyses (PCA and OPLS-DA) support the robustness and reproducibility of the detected metabolic patterns and have yet to be confirmed by analyzing specimens from other resources. Our study provides complementary phytochemical evidence that supports the functional components of cruciferous leaves to corroborate their utilization as functional foods or further in nutraceuticals.

## Materials and methods

### Plant material

Six cruciferous leaves viz. *Brassica oleracea* (cabbage), *B. oleracea* var. *Italica* (broccoli), *B. oleracea* var. *oleracea* (cauliflower), *B. rapa* (turnip), *Raphanussativus L.* (radish), and *Nasturtium officinale* (watercress) were collected from local farm in Qualuob, El-Qualuobia governorate (30.2220° N, 31.3084° E), Egypt, during October and November 2019. The botanical identification of the plants was confirmed by Prof. Dr. Rim Hamdy, Botany Department, Faculty of Science, Cairo University, Egypt. Fresh leaves were lyophilized and kept in airtight containers at −80 °C till preparation for UPLC-MS/MS analysis. Metabolite extraction and LC-MS analysis were conducted in 2020. Three biological replicates were analyzed for each sample. A voucher specimen was deposited at the College of Pharmacy Herbarium, Cairo University, Cairo, Egypt.

### Chemicals and reagents

Acetonitrile and formic acid (LC-MS grade) were obtained from J. T. Baker (The Netherlands); Milli-Q water was used for UHPLC analysis. Chromoband C18 (500 mg, 3 mL) cartridge was purchased from Macherey-Nagel (D¨uren, Germany). All other chemicals and standards were purchased from Sigma-Aldrich (St Louis, MO, USA).

### Preparation of leaf extracts for UHPLC-MS analysis

The extraction of the cruciferous leaves was performed as previously described^41^. Briefly, freeze dried leaves were ground with a pestle in a mortar, with 2 g of each leaf freeze dried powder homogenized with 5mL 100% MeOH containing 10 mg mL^− 1^ umbelliferone (an internal standard used for retention time alignment of UHPLC-MS features) using a Turrax mixer (11 000 rpm) for 20 s periods with recession time of 1 min. Extracts were then vortexed vigorously and centrifuged at 3000 g for 30 min to remove plant debris. For solid phase extraction, 500 µL were aliquoted and loaded on a (500 mg) C18 cartridge, which was preconditioned with methanol and water. Samples were then eluted using 3 µL 70% MeOH and 3 µL 100% MeOH, and eluents were evaporated to dryness under a gentle nitrogen stream. The obtained dry residue was re-suspended in 500 µL 100% methanol for further UHPLC-MS analysis.

### High-resolution UPLC-MS/MS analysis

The analytical conditions for high-resolution UHPLC-MS/MS were employed as previously described^41^. The UHPLC analysis was performed on an Acquity UHPLC System (Waters) equipped with an HSS T3 column (100–1.0 mm, particle size 1.8 mm; Waters). The analysis was carried out by applying the following binary gradient at a low rate of 150 mL min^− 1^: 0–1 min, isocratic 95% A (water/formic acid, 99.9/0.1 [v/v]), 5% B (acetonitrile/formic acid, 99.9/0.1 [v/v]); 1–16 min, linear from 5 to 95% B; 16–18 min, isocratic 95% B; and 18–20 min, isocratic 5% B. The injection volume was 3.1 mL (full loop injection). Eluted compounds were detected from m/z 90 to 1000 using a MicroTOF-Q hybrid quadrupole time-of flight mass spectrometer (Bruker Daltonics) equipped with an Apollo- II electrospray ion source in negative and positive (deviating values in brackets) ion modes using the following instrument settings: nebulizer gas, nitrogen, 1.4 (1.6 bar); dry gas, nitrogen, 6.l min^− 1^, 190 °C; capillary, _5000 V (+ 4000 V); end plate offset, 500 V; funnel 1 RF, 200 Vpp; funnel 2 RF, 200 Vpp; in-source CID energy, 0 V; hexapole RF, 100 Vpp; quadrupole ion energy, 5 eV (3 eV); collision gas, argon; collision energy, 7 eV (3 eV); collision RF, stepping 150/350 Vpp (200/300Vpp), (timing 50/50); transfer time, 58.3 ms; prepulse storage, 5 ms; pulser frequency, 10 kHz; and spectra rate, 3 Hz. Internal mass calibration of each analysis was performed by infusion of 20 mL 10 mM lithium formate in isopropanol: water, 1 : 1 (v/v), at a gradient time of 18 min using a diverter valve. For Auto-MS/MS analysis, precursor ions were selected in Q1 with an isolation width of _3–10 Da and fragmented at collision energies of 15–70 eV using argon as a collision gas. Product ions detection was performed using the same settings as above, but with funnel 2 RF 300 Vpp in negative mode. Metabolites were characterized by their retention times relative to external standards, accurate MS, and the domino MS/MS spectra in comparison to our in-house database, phytochemical dictionary of natural products database, and reference literature. Levels of annotation were reported according to the guidelines of the metabolomics society compound identification work group at their annual meeting (Brisbane, Australia)^42^. Pattern isotopic similarity is already used in formula prediction by Bruker compass program. Metabolite annotation was accepted only when isotopic pattern fit exceeded 85%, and the observed mass error was below 10 ppm; few signals with mass deviation > 10 ppm were manually re-evaluated and excluded when confidence was low. Each crucifer species was represented by three independent biological replicates, which were extracted and analyzed separately to ensure reproducibility and robust statistical comparison.

### MS data processing for multivariate data analysis

Features extraction of the examined cruciferous leaves MS files was performed using XCMS package under R 2.9.2 environment. The script employed for peak detection, alignment, and grouping of the extracted ion chromatograms (**Suppl. Code S1**) follows the exact steps described in ^41^. Normalization and Pareto scaling of the features prior to modelling were performed prior to multivariate models i.e., PCA and OPLS-DA using SIMCA-P Version 13.0 (Umetrics, Umea, Sweden).

## Supplementary Information

Below is the link to the electronic supplementary material.

Supplementary Material 1

## Data Availability

All data generated or analyzed during this study are included in this published article [and its supplementary information files].
