# Supplementary material for: Unveiling crucifer metabolomes via UPLC-HRMS/MS and chemometric analysis of edible and non-edible varieties
Source: Sci Rep. 2025 Dec 8;15:43481. doi: 10.1038/s41598-025-29178-w (PMC12696057; doi:10.1038/s41598-025-29178-w)
Supplement: Supplementary file 1 — Supplementary Material 1 [file 41598_2025_29178_MOESM1_ESM.docx]

**Unveiling Crucifer Metabolomes via UPLC-HRMS/MS and Chemometric Analysis of Edible and Non-Edible Varieties**

**Eman M. Kabbash^1a^, Mostafa H. Baky^2a^, Ahmed Serag^3^, Steffani Doll^4^, Mohamed A. Farag^5^***

*^1^ Phytochemistry Department, Egyptian Drug Authority (Former; National Organization for Drug Control and Research), Giza 12622, Egypt*

*^2^ Department of Pharmacognosy, Faculty of pharmacy, Egyptian Russian University, Badr city, 11829, Cairo, Egypt.*

*^3^Pharmaceutical Analytical Chemistry Department, Faculty of Pharmacy, Al-Azhar*

*University, Cairo, 11751, Egypt*

*^4^ Department of Stress and Developmental Biology, Leibniz Institute of Plant Biochemistry, Weinberg 3, D-06120 Halle (Saale), Germany*

*^5^Pharmacognosy Department, College of Pharmacy, Cairo University, 11562 Cairo, Egypt.*

***^a^: Equal contribution***

⁎ Corresponding author at: Cairo University, College of Pharmacy, Department of Pharmacognosy, Egypt.

E-mail addresses: [mohamed.farag@pharma.cu.edu.eg](mailto:mohamed.farag@pharma.cu.edu.eg) (M.A. Farag).

**Fig. S1:** Tandem MS spectra for structural isomer peaks (40, 41 and 99) kaempferol-*O*- sophoroside–di-*O-*hexoside, and Quercetin-*O*-sophoroside -*O*-hexosyl-deoxyhexoside and Isorhamnetin-*O*-pentosyl-tri-*O*-hexoside [M-H]^-^ 933.2563 C_39_H_49_O_26._

[M-H]^-^

[M-H-162 (C_9_H_6_O_3_]^-^

[M-H-162 (C_9_H_6_O_3_)-162]^-^

[301]^-^

[M-H-162 (C_9_H_6_O_3_)-162-308]^-^

**Fig. S2:** Tandem MS spectrum forpeak (36) Quercetin-*O*-caffeoyldeoxyhexosyl–tri-*O*- hexoside[M-H]^-^ 1095.2897 C_48_H_55_O_29_^-^

[M-H]^-^

[M-H-132-176]^-^

[M-H-132]^-^

[M-H-132-176-132]^-^

**Fig. S3:** Tandem MS spectrum forpeak (80) Kaempferol- *O*-feruloyl-di-*O*-pentoside [M-H]^-^ 725.1912 C_48_H_55_O_29_^-^

[SO_4_H]-

[M-H]^-^

195 [M-H-64-162]^-^

358 [M-H-64 (CH_3_SO)loss of methyl sulfoxide]^-^

**Fig. S4:** Tandem MS spectrum for peak (14) Glucoiberin [M-H]^-^ 422.0228 C_11_H_19_NO_10_S_3_^-^

[M-H]^-^

[SO_4_H]-

[M-H-80 (SO_3_)-2H]^-^

**Fig. S5:** Tandem MS spectrum for peak (15) Gluconapin [M-H]^-^ 372.0422 C_11_H_18_NO_9_S_2_^-^

**
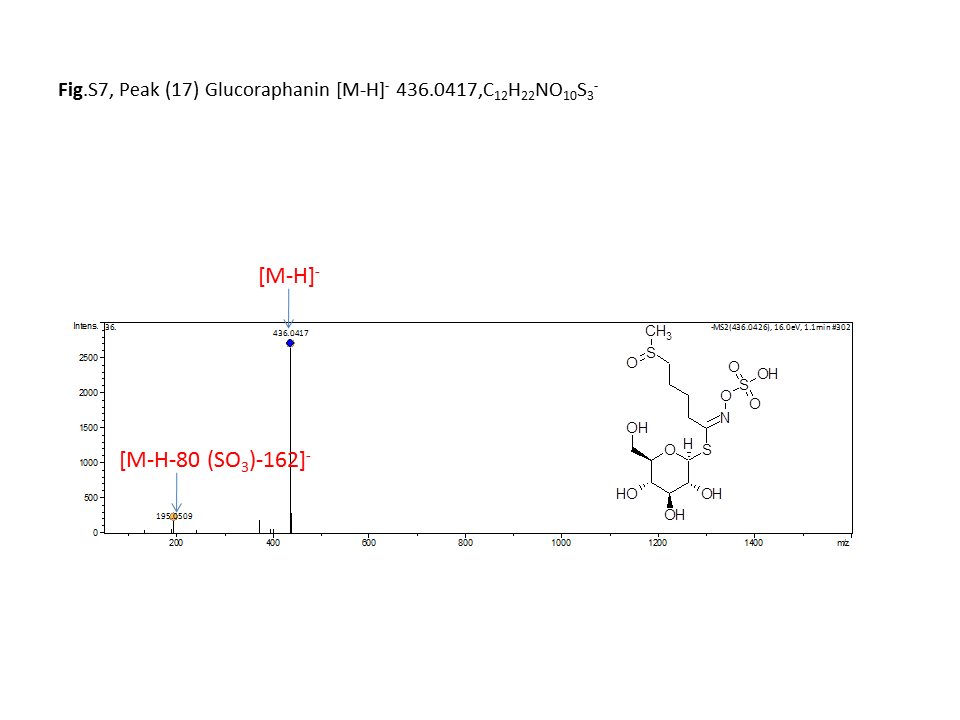
**

**Fig. S6:** Tandem MS spectrum for Peak (16) Glucoraphanin [M-H]^-^ 436.0417,C_12_H_22_NO_10_S_3_^-^

[M+H]^+^

[M+H-324]^+^

[M+H-324-324]^+^

**Fig. S7:** Tandem MS spectrum for Peak (39) Cyanidin-*O*-tetra-hexoside[M+H]^+^ 935.2672 (C_39_H_51_O_26_^+^)^+^]

[M-H]^-^

[M-H-288]^-^

[M-H-288-288]^-^

**Fig. S 8:** Tandem MS spectrum for Peak (128) Gallocatechin-(epi)catechin-( epi)catechin [M-H]^-^ 881.4929, C_45_H_37_O_19_^-^

[M-H]^-^

[M-H-279]^-^

566.3446[M-H-253]^-^

linoleic acid C_18_H_31_O_2_]^-^

[M-H]^-^

[M-H-256]^-^

[M-H]^-^

[M–C16:0+H]^–^

539 [M–C18:3+H]^–^

297 [539-243 (C_6_H_11_O_8_S)]^–^

**Fig. S9:** Tandem MS spectra for identified sulfolipids peak 142 [(M-H)- *m/z* 817.424 (C_43_H_77_O_12_S^-^)^-^for SQDG (18:3/16:0),and peak 143 [(M-H)- *m/z* 819.436 (C_43_H_79_O_12_S^-^) for SQDG (18:1/16:1)


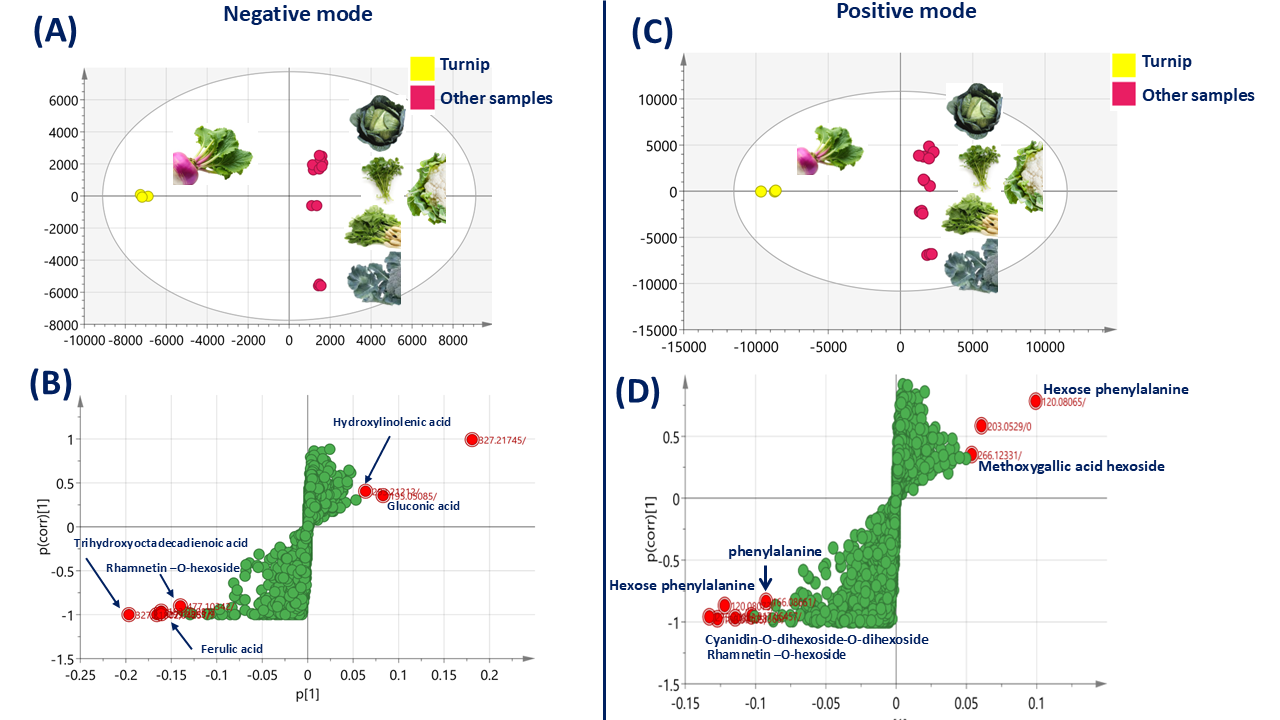


**Fig.** **S10.** Supervised OPLS-DA score plot derived from modeling turnip versus other 5 plantsboth negative ion (A) and positive ion mode (C). The respective loading S-plots showing the covariance p [1] against the correlation p(cor) [1] of the variables of the discriminating component of the OPLS-DA model are depicted in (**B**) and (**D**), respectively.


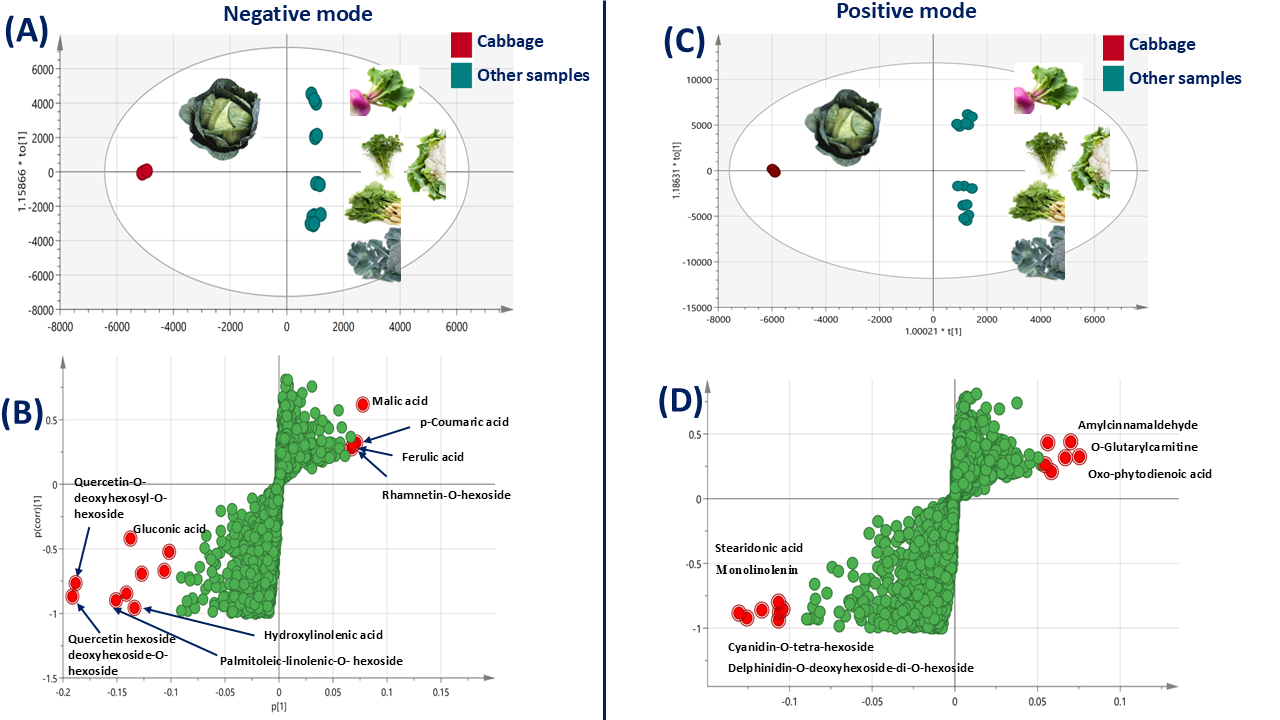


**Fig. S11.** Supervised OPLS-DA score plot derived from modeling cabbage versus other 5 plants both negative ion (A) and positive ion mode (C). The respective loading S-plots showing the covariance p [1] against the correlation p(cor) [1] of the variables of the discriminating component of the OPLS-DA model are depicted in (**B**) and (**D**), respectively.


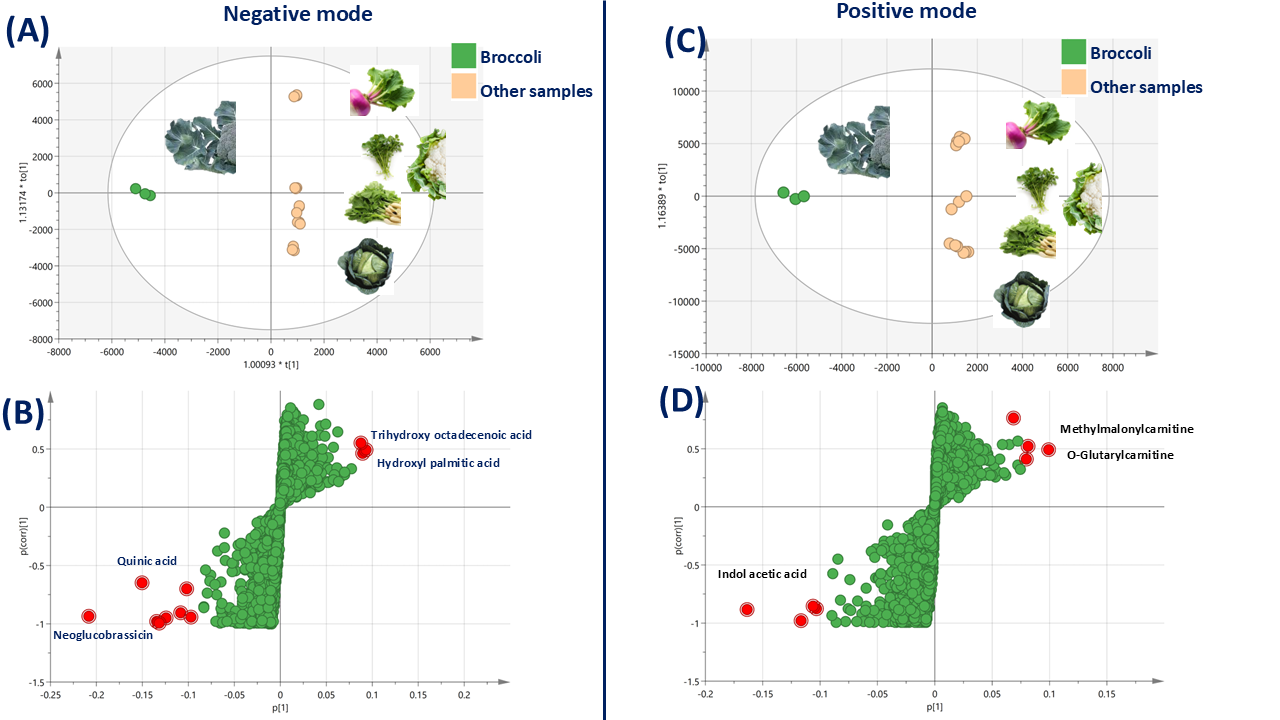


**Fig. S12.** Supervised OPLS-DA score plot derived from modeling broccoli versus other 5 plants both negative ion (A) and positive ion mode (C). The respective loading S-plots showing the covariance p [1] against the correlation p(cor) [1] of the variables of the discriminating component of the OPLS-DA model are depicted in (**B**) and (**D**), respectively.


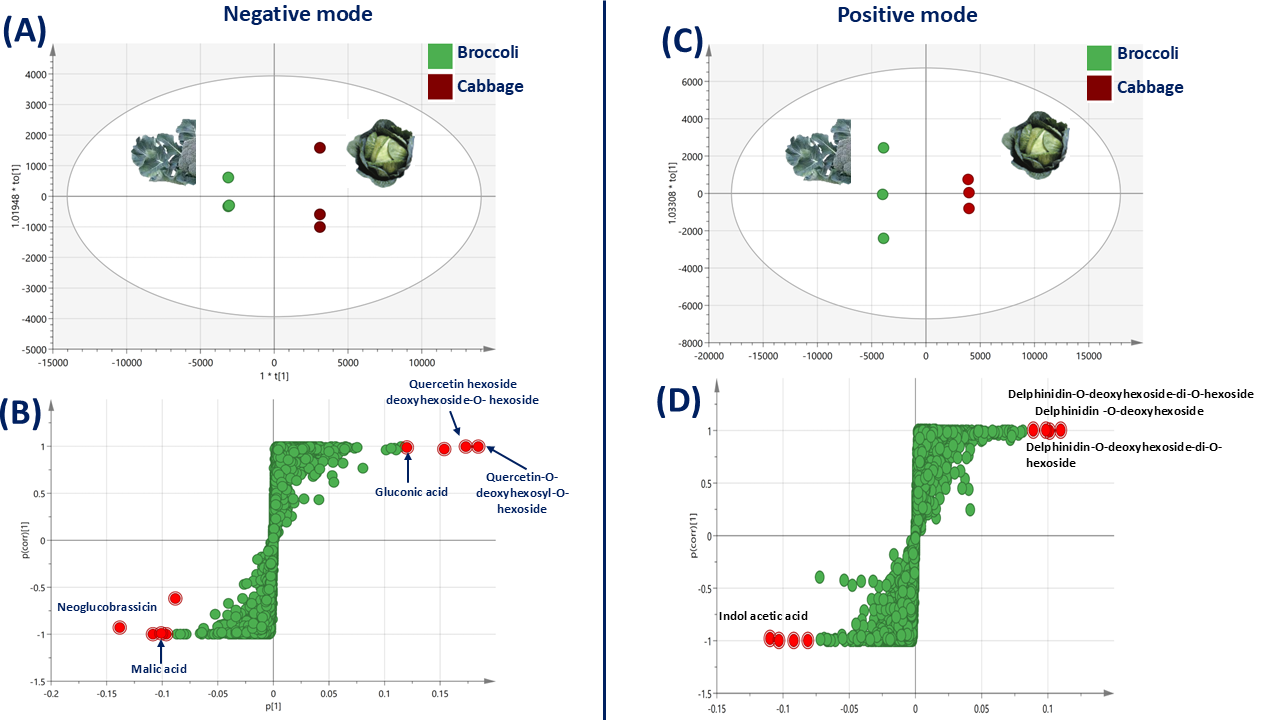


**Fig. S13.** Supervised OPLS-DA score plot derived from modeling broccoli versus cabbage for both negative ion (A) and positive ion mode (C). The respective loading S-plots showing the covariance p [1] against the correlation p(cor) [1] of the variables of the discriminating component of the OPLS-DA model are depicted in (**B**) and (**D**), respectively.


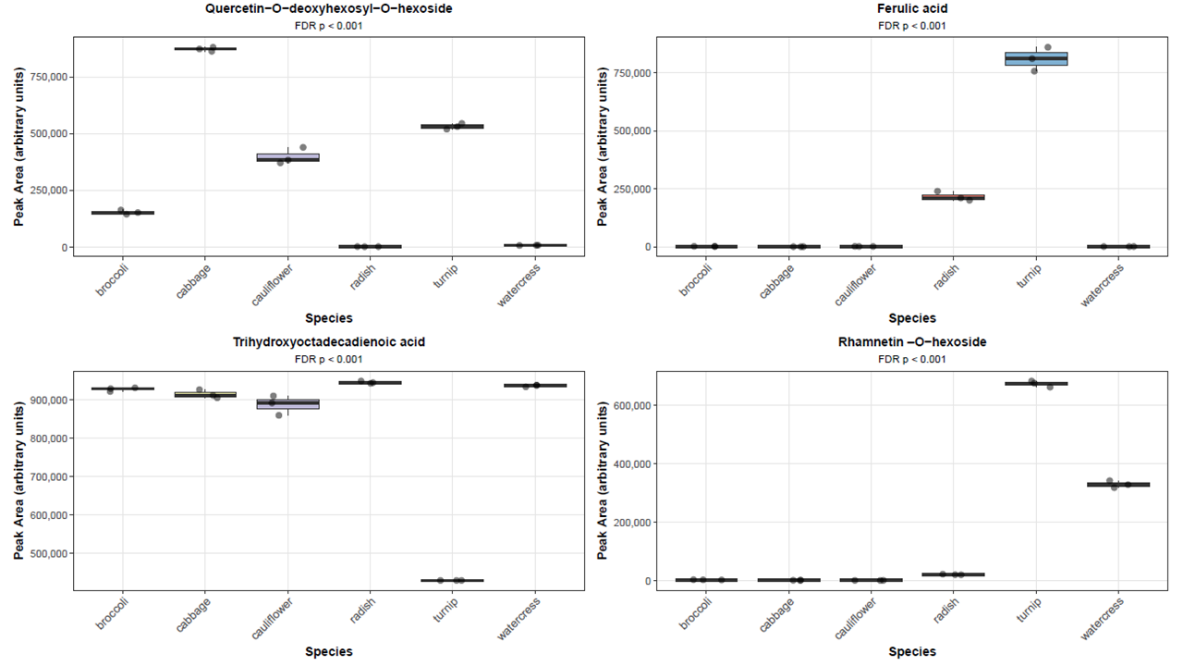

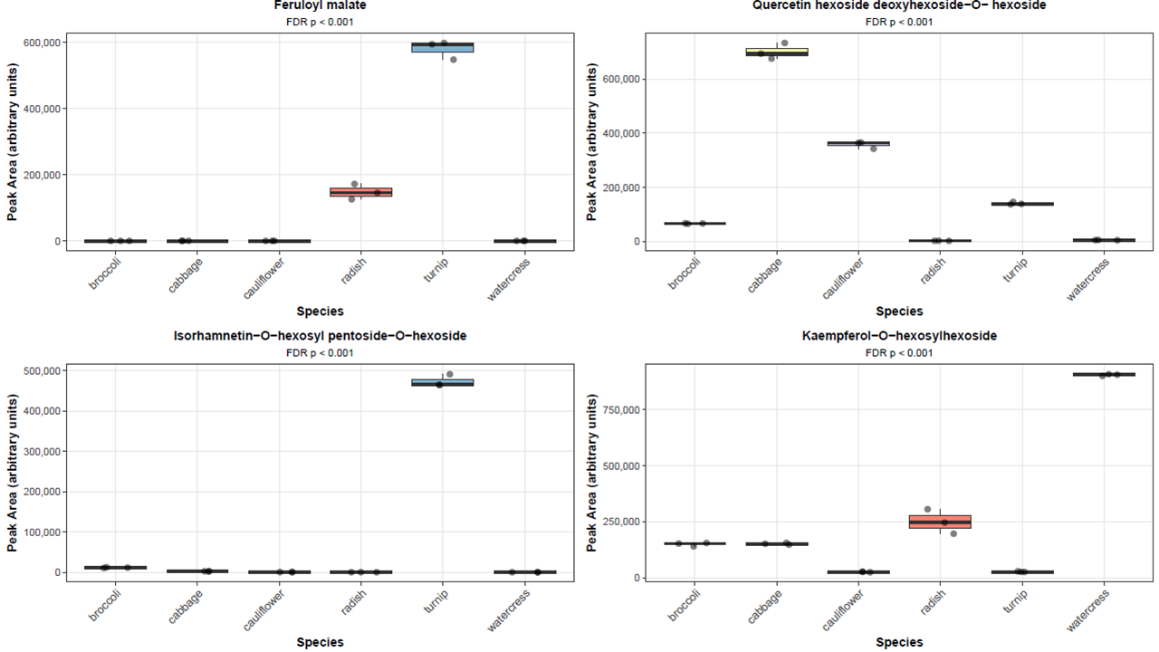

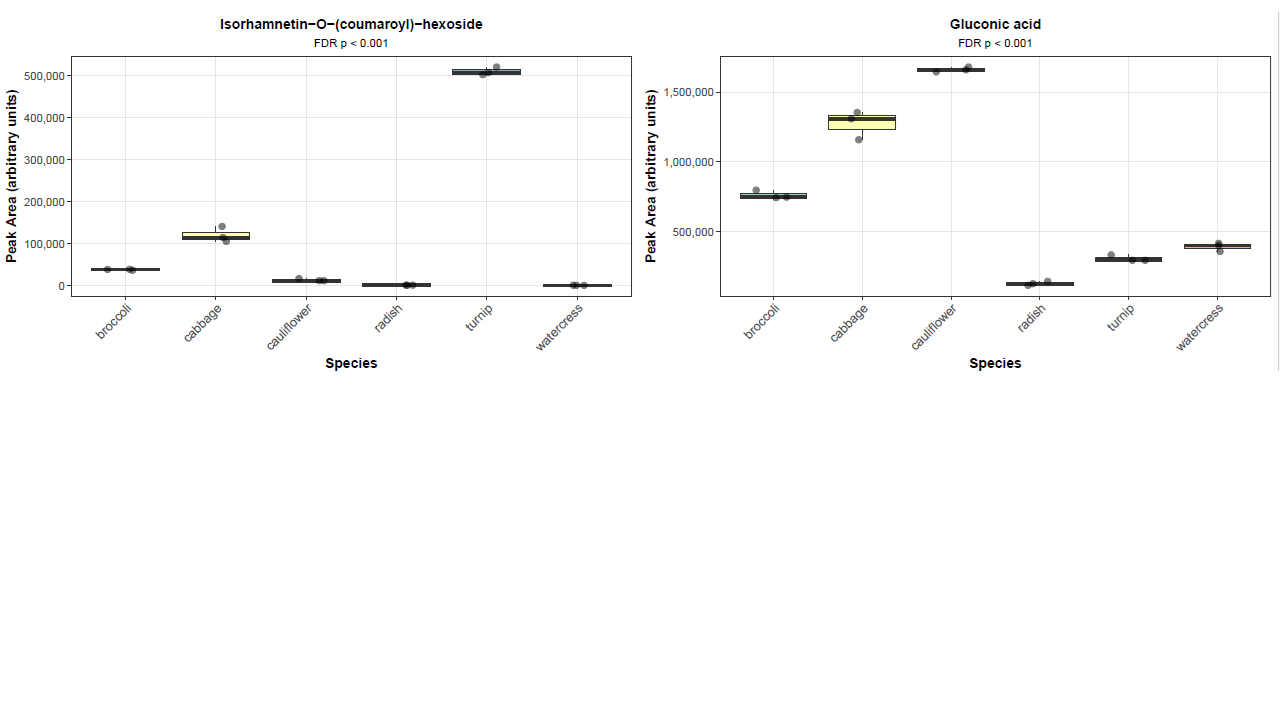


**Figure S14.** Species-specific variation in the relative abundance of marker metabolites identified in cruciferous vegetables detected by UPLC-MS/MS in negative ionization mode. Boxplots represent normalized peak areas (arbitrary units) of the most discriminant metabolites across six species broccoli, cabbage, cauliflower, radish, turnip, and watercress. Each box displays the interquartile range (IQR), with median values indicated by horizontal lines. Significant interspecies differences were confirmed by one-way ANOVA with FDR-adjusted p < 0.001. Notably, quercetin, isorhamnetin, kaempferol, and rhamnetin glycosides showed pronounced species-specific accumulation patterns, whereas ferulic and feruloyl malate represented key phenolic acid markers. Organic acids such as gluconic acid further contributed to species discrimination, reflecting distinct metabolic signatures among cruciferous taxa.


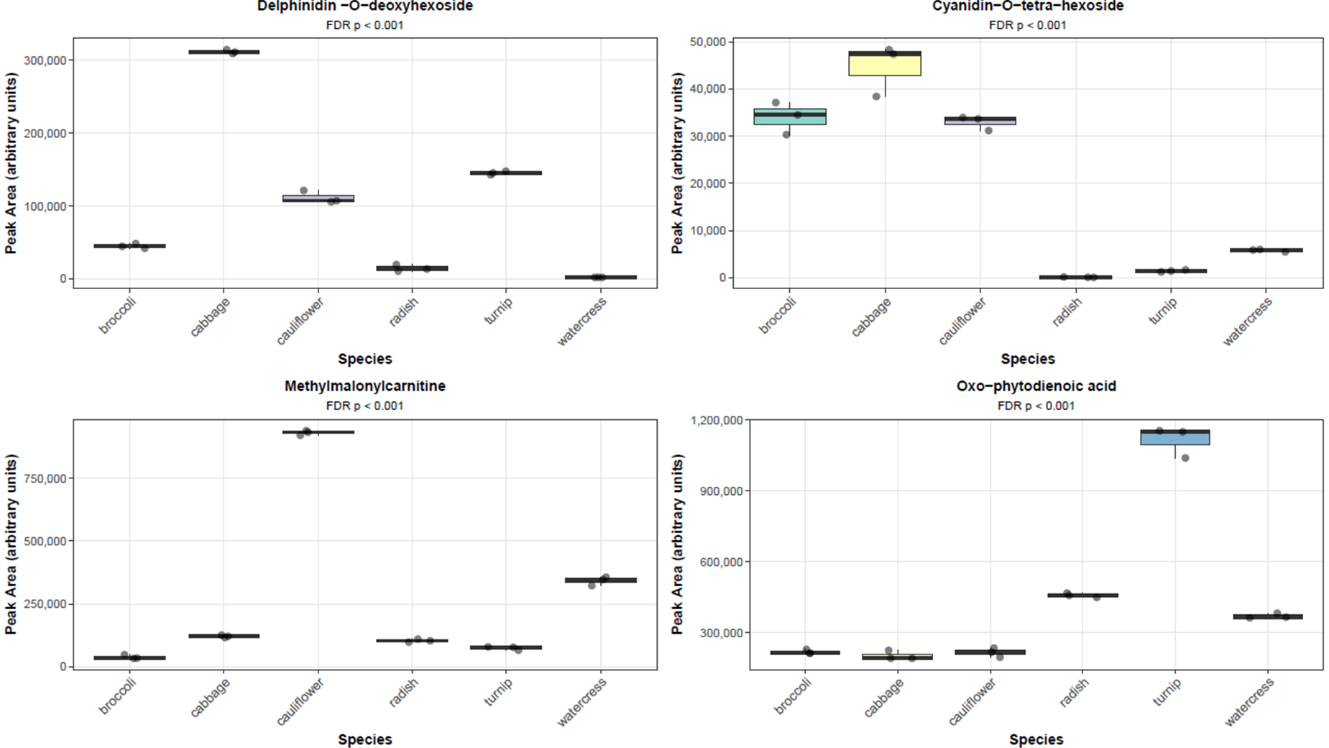

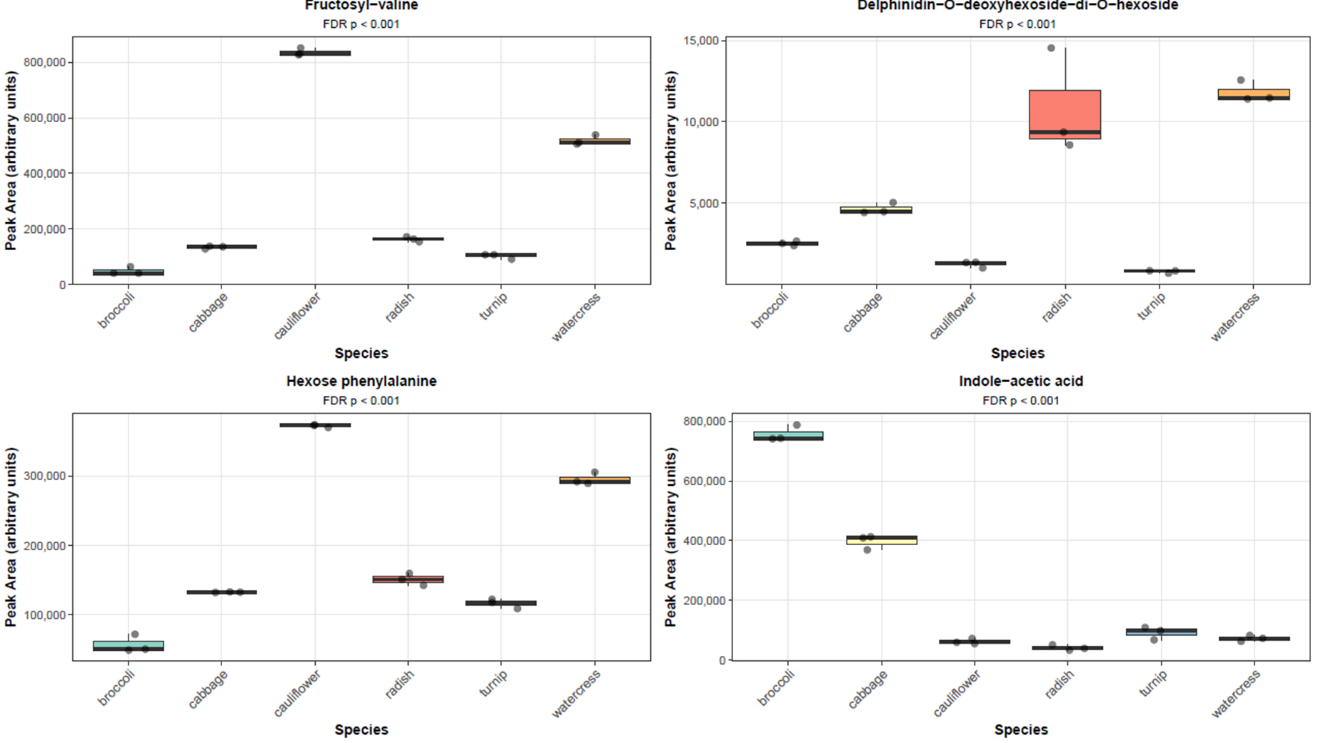

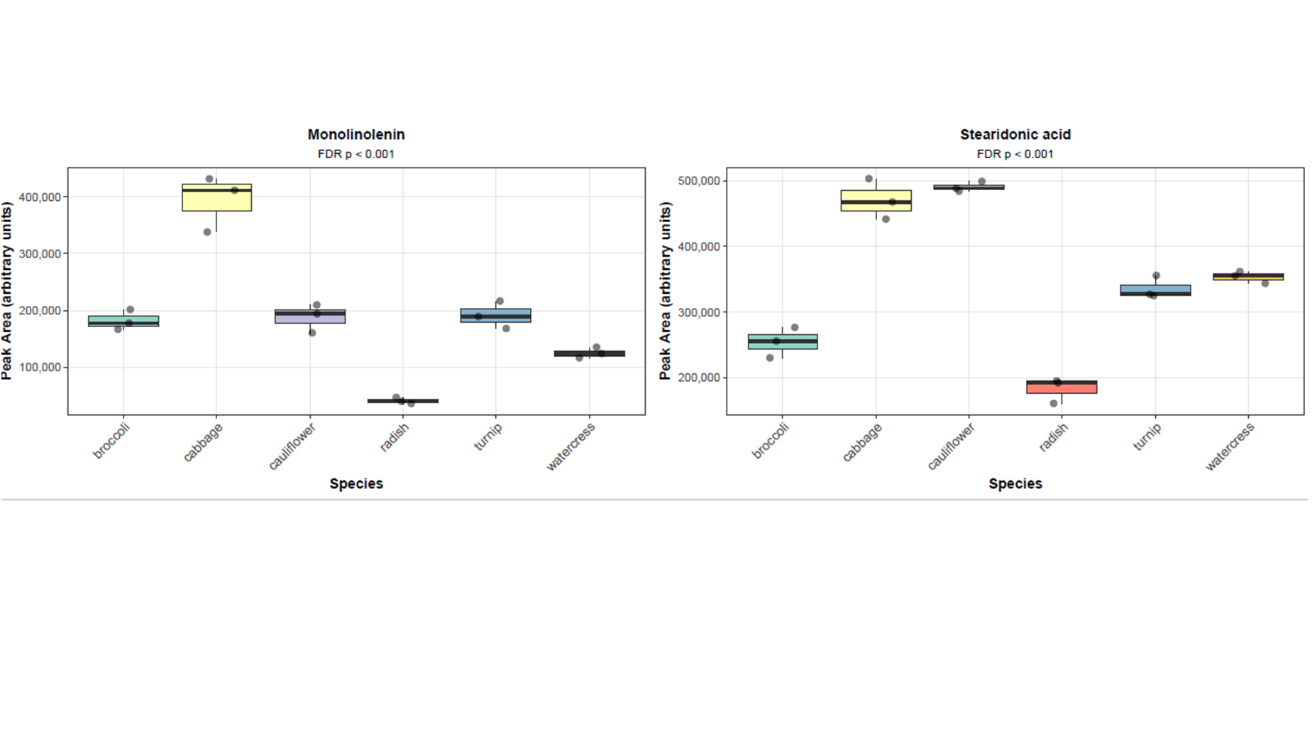


**Figure S15.** Species-specific variation in the relative abundance of marker metabolites identified in cruciferous vegetables detected by UPLC-MS/MS in positive ionization mode. Boxplots depict normalized peak areas (arbitrary units) of the most discriminant metabolites across six species broccoli, cabbage, cauliflower, radish, turnip, and watercress. Each box represents the interquartile range (IQR) with median values shown as horizontal lines. One-way ANOVA with FDR-adjusted p < 0.001 confirmed significant interspecies variation. Anthocyanin derivatives such as delphinidin-*O*-deoxyhexoside and cyanidin-*O*-tetra-hexoside exhibited the highest species specificity, while methylmalonylcarnitine, oxo-phytodienoic acid, and fructosyl-valine indicated differences in acylcarnitine, oxylipin, and amino acid metabolism. Together, these metabolites highlight distinct primary and secondary metabolic adaptations among cruciferous taxa.

| **Table S1. Quantitative analysis of marker metabolites identified in cruciferous vegetables detected in UPLC-MS/MS analysis in negative mode** | | | | | | | |
| --- | --- | --- | --- | --- | --- | --- | --- |
| **Metabolite** | **broccoli** | **cabbage** | **cauliflower** | **radish** | **turnip** | **watercress** | **FDR p-value** |
| Ferulic acid | 2580 ± 227 | 1411 ± 54 | 2502 ± 138 | 217388 ± 11720 | 808683 ± 29727 | 1912 ± 113 | < 0.001 |
| Feruloyl malate | 185 ± 0 | 247 ± 62 | 185 ± 0 | 147918 ± 13368 | 580273 ± 16202 | 185 ± 0 | < 0.001 |
| Gluconic acid | 762017 ± 17232 | 1273019 ± 59021 | 1660662 ± 10036 | 128354 ± 8258 | 307343 ± 12835 | 391567 ± 17380 | < 0.001 |
| Isorhamnetin-O-(coumaroyl)-hexoside | 37688 ± 666 | 119867 ± 10685 | 13130 ± 1691 | 754 ± 83 | 509707 ± 5442 | 392 ± 98 | < 0.001 |
| Isorhamnetin-O-hexosyl pentoside-O-hexoside | 11519 ± 519 | 2334 ± 161 | 432 ± 50 | 150 ± 38 | 473386 ± 8828 | 112 ± 0 | < 0.001 |
| Kaempferol-O-hexosylhexoside | 149030 ± 4661 | 151354 ± 2224 | 25738 ± 716 | 249310 ± 31607 | 26663 ± 983 | 904847 ± 2202 | < 0.001 |
| Quercetin hexoside deoxyhexoside-O- hexoside | 65914 ± 492 | 701775 ± 17060 | 357571 ± 7397 | 1793 ± 245 | 140076 ± 2762 | 4391 ± 341 | < 0.001 |
| Quercetin-O-deoxyhexosyl-O-hexoside | 153307 ± 5297 | 872744 ± 5099 | 398272 ± 21066 | 1849 ± 118 | 531976 ± 7309 | 7620 ± 325 | < 0.001 |
| Rhamnetin –O-hexoside | 3384 ± 270 | 1905 ± 235 | 1375 ± 118 | 21393 ± 658 | 673511 ± 6092 | 329726 ± 6858 | < 0.001 |
| Trihydroxyoctadecadienoic acid | 927750 ± 2890 | 914779 ± 6282 | 887532 ± 14756 | 945888 ± 1791 | 429988 ± 0 | 936915 ± 1368 | < 0.001 |
| *Peak area values (mean ± SE) for top 10 significantly different metabolites* | | | | | | | |
| *Values represent arbitrary units from LC-MS analysis* | | | | | | | |
| *FDR = False Discovery Rate adjusted p-value* | | | | | | | |
| *n = 3 biological replicates per species* | | | | | | | |

| **Table S2. Quantitative analysis of marker metabolites identified in cruciferous vegetables detected in UPLC-MS/MS analysis in positive mode** | | | | | | | |
| --- | --- | --- | --- | --- | --- | --- | --- |
| **Metabolite** | **broccoli** | **cabbage** | **cauliflower** | **radish** | **turnip** | **watercress** | **FDR p-value** |
| Cyanidin-O-tetra-hexoside | 33981 ± 1976 | 44696 ± 3158 | 32920 ± 881 | 90 ± 23 | 1430 ± 130 | 5739 ± 160 | < 0.001 |
| Delphinidin -O-deoxyhexoside | 44854 ± 1848 | 311023 ± 1506 | 111096 ± 4915 | 14442 ± 2602 | 144948 ± 1409 | 1953 ± 26 | < 0.001 |
| Delphinidin-O-deoxyhexoside-di-O-hexoside | 2540 ± 78 | 4656 ± 196 | 1257 ± 111 | 10828 ± 1866 | 808 ± 50 | 11813 ± 378 | < 0.001 |
| Fructosyl-valine | 48278 ± 7839 | 133993 ± 2886 | 836984 ± 7575 | 163182 ± 5182 | 101632 ± 5239 | 518835 ± 10193 | < 0.001 |
| Hexose phenylalanine | 57212 ± 7349 | 132666 ± 292 | 372026 ± 1177 | 150929 ± 4871 | 116433 ± 3899 | 295452 ± 5093 | < 0.001 |
| Indole-acetic acid | 757472 ± 15301 | 396731 ± 14161 | 61127 ± 5195 | 40259 ± 5154 | 91011 ± 12345 | 71656 ± 5682 | < 0.001 |
| Methylmalonylcarnitine | 37903 ± 4619 | 120779 ± 2841 | 932184 ± 5383 | 103257 ± 3449 | 73942 ± 4045 | 341703 ± 10088 | < 0.001 |
| Monolinolenin | 182139 ± 10016 | 393309 ± 28437 | 187978 ± 14524 | 41608 ± 3099 | 191542 ± 13729 | 125280 ± 5260 | < 0.001 |
| Oxo-phytodienoic acid | 218938 ± 5268 | 203241 ± 11224 | 216784 ± 11061 | 458827 ± 5194 | 1115557 ± 37635 | 370812 ± 6138 | < 0.001 |
| Stearidonic acid | 254149 ± 13464 | 471170 ± 17892 | 490857 ± 4383 | 182580 ± 11054 | 335926 ± 9911 | 353634 ± 5262 | < 0.001 |
| *Peak area values (mean ± SE) for top 10 significantly different metabolites* | | | | | | | |
| *Values represent arbitrary units from LC-MS analysis* | | | | | | | |
| *FDR = False Discovery Rate adjusted p-value* | | | | | | | |
| *n = 3 biological replicates per species* | | | | | | | |

**Suppl. Code S1.** R script used for extracting the UHPLC-MS features of the examined Cruciferous vegetables

### loading xcms library####

library(xcms)

### Setting working directory and assigning the UHPLC-MS files ####

setwd("F:\\ crucifereae ")

### for Windows

lpath<-"." files <- list.files(lpath, full.names = T, pattern = ". mzXML ", recursive = T)

####Peak detection of the chromatographic features in each sample using “centwave” algorithm#####

xset <- xcmsSet(files=files, method='centWave', ppm=25, peakwidth=c(5,12), snthr=3, verbose.columns=F, scanrange=c(100,1800), prefilter=c(3,500),nSlaves=10)

####Grouping the peaks across all the samples#####

xset_group1 <- group(xset, minfrac = 0.5, bw = 5, mzwid = 1, max = 50)

####Retention time alignment #####

xset_retcor1 <- retcor(xset_group1, plottype ="mdevden", span = 1, missing = 1, extra = 1)

####Regrouping after alignment#####

xset_group2 <- group(xset_retcor1, minfrac = 0.5, bw=2, mzwid=0.05)

####Filling the missing data using the raw data files#####

xset_filled <- fillPeaks(xset_group2)

####Generating the final raw data matrix of the detected EICs#####

values <- groupval(xset_filled, value="into")

####Saving the matrix in a csv file for further processing#####

write.csv(values, file=" Cruciferae_neg_minfrac0.5.csv")
